# Combating Drug Resistance by Exploiting miRNA-200c-Controlled Phase II Detoxification

**DOI:** 10.3390/cancers14225554

**Published:** 2022-11-11

**Authors:** Bianca Köhler, Sviatlana Dubovik, Elisa Hörterer, Ulrich Wilk, Jan Bernd Stöckl, Hande Tekarslan-Sahin, Bojan Ljepoja, Philipp Paulitschke, Thomas Fröhlich, Ernst Wagner, Andreas Roidl

**Affiliations:** 1 Pharmaceutical Biotechnology, Department of Pharmacy, Ludwig-Maximilians-Universität München, D-81377 Munich, Germany; 2 Laboratory of Functional Genome Analysis (LAFUGA), Gene Center, Ludwig-Maximilians-Universität München, D-81377 Munich, Germany; 3 PHIO Scientific GmbH, Esswurmstr. 16, D-81371 Munich, Germany

**Keywords:** cancer, glutathione S-transferase, miR-200c, chemotherapy, resistance

## Abstract

**Simple Summary:**

The resistance formation of cancer cells to chemotherapeutic drugs is one of the main reasons for the failure of cancer therapy. In order to combat drug resistance and improve the efficacy of chemotherapeutic drugs, we studied the role of the multifaceted hsa-miR-200c in different tumor types. We identified hsa-miR-200c as an important player regulating phase II detoxification and thus sensitizing cells to chemotherapeutics and reverse drug resistance. In a xenograft mouse experiment, the mutual expression of hsa-miR-200c and chemotherapeutic treatment led to a regression of tumor size and eventually to the survival of 60% of the mice. These findings highlight hsa-miR-200c both as a potential prognostic marker for chemotherapy and as a novel therapeutic option in cancer therapy.

**Abstract:**

Acquired drug resistance constitutes a serious obstacle to the successful therapy of cancer. In the process of therapy resistance, microRNAs can play important roles. In order to combat resistance formation and to improve the efficacy of chemotherapeutics, the mechanisms of the multifaceted hsa-miR-200c on drug resistance were elucidated. Upon knockout of hsa-miR-200c in breast carcinoma cells, a proteomic approach identified altered expression of glutathione S-transferases (GSTs) when cells were treated with the chemotherapeutic drug doxorubicin. In different hsa-miR-200c expression systems, such as knockout, inducible sponge and inducible overexpression, the differential expression of all members of the GST family was evaluated. Expression of hsa-miR-200c in cancer cells led to the repression of a multitude of these GSTs and as consequence, enhanced drug-induced tumor cell death which was evaluated for two chemotherapeutic drugs. Additionally, the influence of hsa-miR-200c on the glutathione pathway, which is part of the phase II detoxification mechanism, was investigated. Finally, the long-term effects of hsa-miR-200c on drug efficacy were studied in vitro and in vivo. Upon doxycycline induction of hsa-miR-200c, MDA-MB 231 xenograft mouse models revealed a strongly reduced tumor growth and an enhanced treatment response to doxorubicin. A combined treatment of these tumors with hsa-miR-200c and doxorubicin resulted in complete regression of the tumor in 60% of the animals. These results identify hsa-miR-200c as an important player regulating the cellular phase II detoxification, thus sensitizing cancer cells not expressing this microRNA to chemotherapeutics and reversing drug resistance through suppression of GSTs.

## 1. Introduction

Despite important progress in cancer therapy, resistance to anti-cancer drugs constitutes the main obstacle to the successful treatment of advanced tumors [1,2,3]. Although most tumors are chemosensitive at therapy initiation, drug resistance can evolve and patients subsequently suffer from recurrence. Driven by genomic instability, genetic heterogeneity is generated and the subsequent selection of tumor cells surviving the chemotherapeutic treatment fuels the formation of chemoresistance [4]. Acquired resistance comprises multifactorial mechanisms [5,6], such as the reinforcement of drug-detoxifying mechanisms and drug efflux pumps, quantitative and qualitative modification of drug targets, altered regulation of DNA replication or enhanced DNA repair mechanisms and the modulation of apoptosis [7,8]. One of these fundamental mechanisms to augment drug resistance is reducing the intracellular concentration of chemotherapeutic drugs, which leads to less harmful metabolites. These are subsequently eliminated and do not cause damage to the tumor cells. In this regard, the naturally occurring elimination of xenobiotics by phase II detoxification can be exploited by cancer cells [9]. Herein, glutathione S-transferases (GSTs) catalyze the conjugation of glutathione (GSH) to electrophilic xenobiotics resulting in less toxic derivates of the compounds which eventually will be eliminated from the cell and therefore reduce the cellular drug accumulation [9,10,11,12]. GSTs are divided into three major families: cytosolic, mitochondrial (also known as kappa (κ) family) and membrane-bound microsomal GSTs [9,10,11,13]. Mainly involved in phase II detoxification are cytosolic glutathione S-transferases which also form the biggest group of GSTs [9,13,14,15]. These soluble, dimeric enzymes can be divided into seven distinct classes: alpha (A), mu (M), pi (P), sigma (S), omega (O), theta (T), and zeta (Z) [10,14,15]. GSTs frequently contribute to drug resistance by enhancement of their expression [9,13,16]. In this case, GSH-xenobiotic conjugates are formed faster and are actively exported out of the cells, in particular via efflux pumps such as MRP1 and p-glycoproteins [13,15].

Controlling these detoxifying mechanisms is crucial to prevent drug resistance. Lots of evidence has been collected that miRNAs play an important role in this scenario by regulating the translation of different mRNAs [17]. Functionally, miRNAs bind to the three prime untranslated regions (3′UTR) of mRNAs whose expression is subsequently altered at the post-transcriptional level [12,18,19,20]. An interesting microRNA involved in resistance formation is hsa-miR-200c. This multifaceted miRNA belongs to the miRNA-200 family consisting of five members which are derived from two chromosomal locations: hsa-miRNA-200b, -200a and -429 from chromosome 1p36.33 and hsa-miR-200c and -141 from chromosome 12p13.31. Based on their seed sequence, members of miR-200 are assigned to two functional families, hsa-miR-200a, -141 and hsa-miR-200b, -200c, -429 [21]. In our previous studies, we found TrkB, Bmi1, Kras and other oncogenes being controlled by hsa-miR-200c which are important to influence the chemosensitivity of tumor cells [21,22,23,24]. In the case of hsa-miR-200c, also ABC transporters, such as ABCG2, ABCG5, and MDR1 can become inhibited [25]. Whether tumors are sensitive to chemotherapeutic drugs has implications for the outcome of cancer therapy. Clinical data show prolonged survival of breast cancer patients when high levels of hsa-miR-200c were expressed in the tumors [26,27]. Furthermore, hsa-miR-200c plays important roles in the migration, invasion and epithelial-to-mesenchymal transition (EMT) of cancer cells [18,28,29,30,31,32]. EMT, in turn, is also known to alter drug resistance via different pathways, i.e., Wnt and Hedgehog [33,34], or the induction of EMT-inducing transcriptional factors such as Twist, Snail and ZEB [32,35,36].

In this study, we demonstrate for the first time that the 23 nucleotides of hsa-miR-200c control the cellular phase II detoxification, which is a crucial mechanism in drug resistance.

## 2. Materials and Methods

### 2.1. Reagents

Doxycycline hyclate (cat. no. D9891), doxorubicin hydrochloride (cat. no. D1515) and cis-platinum(II)diamine dichloride (cat. no. P4394) were purchased from Sigma-Aldrich (St. Louis, MI, USA). Doxycycline hyclate (DOX) was solved in sterile RNase/DNase-free water. Doxorubicin hydrochloride (DXR) was solved in DMSO or 0.9% sodium chloride solution (Deltamedica, Reutlingen, Germany) for animal experiments. Cis-platinum(II)diamine dichloride (CP) was solved in DMF. Lipofectamine 3000 was purchased from ThermoFisher Scientific (Waltham, MA, USA, cat. no. L3000008). Synthetic hsa-miR-200c was purchased from AxoLabs (Kulmbach, Germany) with the following sequence:

Sense: 5′ UAAUACUGCCGGGUAAUGAUGGA 3′;
Antisense: 5′ UCCAUCAUUACCCGGCAGUAUUA 3′.
The control siRNA duplex with a scrambled sequence was also obtained from AxoLabs:
Sense: 5′ AuGuAuuGGccuGuAuuAGdTsdT 3′;
Antisense: 5′ CuAAuAcAGGCcAAuAcAUdTsdT 3′.

### 2.2. Cell Culture

MDA-MB 231 cells were acquired from DSMZ (Braunschweig, Germany), MDA-MB 231 Tripz 200c and MDA-MB 231 Tripz Ctrl were generated in our lab [37]. All MDA-MB 231 cells were cultured at 37°C and 0% CO_2_ in Leibovitz’s L-15 medium (Sigma-Aldrich) supplemented with 10% fetal calf serum (FCS, Gibco, ThermoFisher Scientific, Hanover Park, IL, USA). MCF7 wildtype cells were acquired from Cell Line Service (Eppelheim, Germany) and cultured at 37 °C and 5% CO_2_ in high glucose DMEM (Sigma-Aldrich) supplemented with 10% FCS (Gibco, ThermoFisher Scientific). The MCF7 KO 200c clones M1, M2 and M3 were generated in our lab as previously described [21] and cultured according to parental MCF7 (wt) cells. MCF7 Tripz 200c sponge cells were also generated in our lab. A549 Tripz 200c and T24 Tripz 200c were generated in our lab and cultured in low glucose DMEM (Sigma-Aldrich) supplemented with 10% FCS (Gibco, ThermoFisher Scientific) at 37 °C and 5% CO_2_. All cells were tested mycoplasm free. Further information on the generation of the cell lines can be found in Appendix A.

### 2.3. Proteomics Sample Preparation

Twenty-four hours after cell seeding (*n* = 3), samples were treated with 5 µM DXR for 6 h. After washing the cells three times with cold PBS, they were lysed with 8 M urea in 400 mM ammonium hydrogen carbonate. Cell lysis was assisted with sonication, followed by homogenization using QIAshredder spin columns (Qiagen, Hilden, Germany). Briefly, 20 µg of protein was incubated for 30 min at a final concentration of 5 mM dithioerythritol (DTE) to reduce disulfide bridges. Cleaved disulfide bonds were then alkylated with iodoacetamide (final concentration 15 mM) for 30 min in the dark. After dilution with water to a concentration of 1 M urea, samples were digested over night with 400 ng porcine trypsin (Promega, Madison, WI, USA) at 37°C. Samples were desalted using C18 spin columns (Pierce, ThermoFisher Scientific) following the manufacturer’s recommended procedure.

### 2.4. Proteomics LC-MS/MS Analysis

1 µg of peptides, dissolved in 15 µL solvent A (0.1% formic acid in water), were injected in an Ultimate 3000 (Thermo Scientific, Waltham, MA, USA) chromatography system and loaded on a capillary trap column (PepMap 100 C18, 100 µm × 2 cm, 5 µM particles, Thermo Scientific). Peptides were subsequently separated at 250 nL/min using an EASY-Spray column (PepMap RSLC C18, 75 µm × 50 cm, 2 µm particles, Thermo Scientific) with a two-step gradient: in the first step, ramping from 5% solvent A to 25% solvent B (0.1% formic acid in acetonitrile) in 160 min, followed by a second ramp from 25% to 40% solvent B in 10 min. MS analysis was performed with a QExactive HF-X mass spectrometer. Using data-dependent acquisition, up to 15 MS/MS spectra per precursor scan were acquired. Precursor spectra were acquired at a resolution of 60,000 (mass-range: 350–1600) and MS/MS spectra at a resolution of 15,000. MS data were deposited in PRIDE. Accessibility possible upon request.

### 2.5. LC-MS/MS Data Analysis

Mass spectrometry (MS) data were processed with MaxQuant (version: 1.6.5.0) using the human subset from Swiss-Prot (downloaded 2 May 2020) and the MaxQuant common contaminants database. The false discovery rate was set to be <0.01 and proteins that were only identified by site or potential contaminants were filtered out. For data analysis and evaluation, Perseus (version 1.6.5.0) was used [38]. For label-free quantification (LFQ), samples were grouped and filtered for at least 70% valid values per group. Missing values were imputed from a normal distribution (width, 0.3; down-shift, 1.8). To test for differentially abundant proteins, a modified two-sided Welch’s *t*-test (s0 = 0.1) was employed. Multiple testing correction was performed with the permutation-based approach included in Perseus, resulting in a false discovery rate of <0.05. The gene set enrichment analysis was carried out using GSEA V4.0.2 (Broad Institute, Cambridge, MA, USA). As gene sets, the gene ontology database as well as the pathway databases REACTOME and KEGG were employed.

### 2.6. Generation of 3′UTR GSTM3 Mutations in pISO

The 3′UTR fragment of GSTM3 was amplified and cloned downstream of a luciferase reporter system in the pISO vector (Addgene plasmid #12178). Amplification of the 3′UTR fragment of GSTM3 was performed with the following primers (Sigma-Aldrich):

5′-TTACAGAGCTCATCCTGTCCGTAAGGGGTCA-3′ (forward),

5′-TGTAATCTAGAAGTCTGAAATACTGCCTTTATCAC-3′ (reverse).

To generate part or full mutation of the binding site for hsa-miR-200c-3p at the 3′UTR of GSTM3, the reverse primers (Sigma-Aldrich) listed below that contained nucleotide mismatches were used:

Mut 1     5′-TGTAATCTAGAAGTCTGAAACACTGCCTTTATCAC-3′

Mut 2     5′-TGTAATCTAGAAGTCTGAAATACATCCTTTATCAC-3′

Full mutation   5′-TGTAATCTAGAAGTCTGATCACAATCCTTTATCAC-3′

Putative miRNA-mRNA seed-site interactions for hsa-miR-200c-3p were analyzed in silico using TargetScan [39,40,41]. Sequences of 3′UTRs and the predicted site types of the different GSTs were also adapted from TargetScan.

### 2.7. Co-Transfection and Luciferase-Reporter Assay

For co-transfection of pDNA and hsa-miR-200c, MCF7 KO 200c M2 cells were seeded in a 6-well plate. Then, 24 h after seeding, cells were transfected with 6 µL Lipofectamine 3000 and 75 pmol hsa-miR-200c or scrambled control siRNA and 1 µg pDNA per well at the same time. After 48 h of incubation, cells were lysed, and a luciferase assay was performed using standard protocol [42].

### 2.8. RNA-Lysis and Purification

Cells were induced with 5 µg/mL DOX for 72 h where necessary. Subsequently, cells were treated with the indicated concentration of DXR (or solvent), when 80% of confluency was achieved. After appropriate incubation with the treatment agent, cells were harvested and purified using the Micro RNA Kit (peqGOLD Micro RNA Kit Safety-Line, cat. No. 732-3088, VWR, Darmstadt, Germany) following the manufacturer’s protocol. For RNA lysis of tumor samples, 20 mg of tumor tissue per animal was homogenized in the appropriate lysing buffer (Micro RNA Kit, Safety-Line, peqlab-VWR), using MP Biomedicals™ Lysematrix D, (ThermoFisher Scientific, cat. No. 11432420) in a homogenizer following manufacturer’s protocol (peqGOLD Micro RNA Kit, cat. No. 732-3088, VWR).

### 2.9. cDNA Synthesis

Following RNA purification, cDNA was synthesized, according to the manufacturer’s protocol, using 1 µg RNA. QScript cDNA Synthesis Kit (Quantabio, Beverly, MA, USA) for mRNA or qScript™ microRNA cDNA Synthesis Kit for miRNA (Quantabio) were used.

### 2.10. Quantitative Real-Time Polymerase Chain Reaction (qRT-PCR)

Messenger RNA expression values were analyzed using qRT-PCR. The LightCycler 480 (Roche, Basel, Switzerland), the Universal Probe Library (UPL, Roche Diagnostics Germany, Mannheim, Germany) and LightCycler 480 Probes Master (Roche Diagnostics Germany) were used. The sample composition was described previously [43]. Briefly, 5 µL cDNA with a 1:10 dilution, after cDNA synthesis, were added per well. Primer probe pairs are specified in Table A1.MiRNA expression was also analyzed using qRT-PCR. The sample mixture was prepared according to the manufacturer’s protocol (qScript™ microRNA cDNA Synthesis Kit for miRNA, Quantabio), and 10 µL microRNA cDNA (1:50 dilution after cDNA synthesis) was used per well.

The 2^−ΔCt^ or the 2^−ΔΔCt^ method was used for quantification. GAPDH or hsa-miRNA-191 were utilized as housekeepers. 

### 2.11. Protein Lysis and Western Blot

Cells were lysed after individual treatment with cell lysis buffer containing cell culture lysis 5 × reagent (Promega, Madison, WI, USA, cat. no. E1531), cOmplete™, Mini, EDTA-free Protease Inhibitor Cocktail (Roche, cat. no. 11836153001) and Sodium orthovanadate (Sigma-Aldrich, cat. no. S6508). Briefly, 30 μg protein (determined via BCA assay, manufacturer’s protocol, ThermoFisher Scientific, cat. no. #23228 and #23224) per sample was analyzed. PVDF-membranes were blocked with NET-gelatin prior to overnight incubation at 4 °C with GSTM3-antibody (ThermoFisher Scientific, cat. no. PA5-57191) solution (1:1000) in NET-gelatin or GAPDH-antibody (1:10,000, loading control, Sigma-Aldrich, cat. no. G9545-100 UL). After washing, membranes were incubated in horseradish peroxidase-conjugated anti-rabbit secondary antibody (goat anti-rabbit-hrp, cat. no. PI-1000, Vector Laboratories, Newark, CA, USA) at room temperature. Desired proteins were detected utilizing enhanced chemiluminescence (Lumi-Light^PLUS^ Western Blotting Substrate, Roche, cat. no. 12015196001) on X-ray films (Amersham Hyperfilm ECL, GE Healthcare, VWR cat. no. 28-9068-39, Darmstadt, Germany). For quantification contrast ratios were analyzed using ImageJ 1.53e. Briefly described, lane profile plots for GSTM3 and GAPDH and corresponding peak areas were measured. Subsequently, all sample areas and all loading-control areas were separately displayed as percent of the total size of measured peaks within the same protein. The relative density of the areas was calculated by ratio determination of the percent of the sample to percent control (always the first sample line of a blot, the corresponding value is 1.0). Final adjusted density values were calculated as the ratio of relative sample density to the relative loading-control density of each sample. Samples are normalized to the control sample (first sample line of a blot).

### 2.12. Analysis of Total Glutathione Using the GSH/GSSG-Glo Assay

Glutathione and GSSG were measured using the GSH/GSSG-Glo assay (Promega Madison, WI, USA). Cells were treated with either 0.1 µM DXR for the MCF7 and 0.6 µM DXR or 50 μM CP for MDA-MB 231 in a 96-well format. Tripz-constructs were pre-induced for 72 h with 5 µg/mL DOX before DXR treatment. The assay was performed according to the manufacturer’s protocol. 

### 2.13. Evaluation of Cell Death Using Propidium Iodide Assay 

Cells were treated with DXR or the appropriate solvent control for 72 h prior to FACS measurement. MCF7 cells were treated with 0.1 µM DXR, MDA-MB 231 cells with 0.6 µM DXR or 50 μM CP, A549 cells with 0.06 µM DXR or 8.5 μM CP and T24 cells with 0.05 µM DXR or 3.5 μM CP. The inducible constructs were 24 h pre-induced with 5 µg/mL DOX before DXR treatment. Propidium iodide (PI) staining was performed by harvesting the floating cells as well as the detached cells after trypsinization. Cells were incubated for three hours with intermittent shaking at 4 °C with 50 µg/mL PI solution. Subsequently, the cell cycle was analyzed using FACS. Evaluation of the cell cycle phases was performed using FlowJo 7.6.5.

### 2.14. Analysis of Long-Term Effects of hsa-miR-200c In Vitro Using the Cellwatcher System

The same number of MDA-MB 231 Tripz 200c cells was seeded and either induced with 5 µg/mL DOX or not. The medium was changed every 48–72 h (containing DOX or not). When the cells reached a confluency of 80%, they were treated with 0.1 µM of DXR for 48 h followed by medium change. Confluency was monitored over a time of 800 h using the PHIO cellwatcher (PHIO scientific, Munich, Germany). 

After termination, cells were washed with ice-cold PBS, fixed with methanol for 10 min and placed on ice, for colony formation assay. Briefly, 0.5% crystal violet in 25% methanol was applied at room temperature for 10 min to the cells and subsequently washed with water.

### 2.15. In Vivo Xenograft Studies of hsa-miR-200c as Genetic Biomarker

Five million human MDA-MB 231 Tripz 200c cells were injected s.c. into the left flank of 6-week-old female NMRI-nu mice (Janvier, Le-Genest-St-Isle, France). Tumor growth was monitored using caliper measurement (a × b^2^ /2; a = longest side of the tumor; b = widest side vertical to a) [44]. Animal well-being and weight were monitored throughout the whole experiment. Mice were either fed continuously with a regular diet or with doxycycline containing diet (+ 625 mg/kg doxycycline, sniff Spezialdiäten, Soest, Germany, cat. no. A115 D70624) depending on the animal study. 

Initial tumor growth was monitored from the day of tumor cell injection till the day when the first animal of each diet group reached the tumor volume of 150 to 200 mm^3^. 

At this tumor size, mice were randomized into four groups depending on the subsequent treatment regime for the animal experiment “(I) Treatment of hsa-miR-200c positive and negative tumors”. Each diet group was subdivided into a group of animals with either DXR treatment (5 mg/kg) or control treatment with saline (0.9% sodium chloride solution). For the animal experiment “(II) Single or double treatment of hsa-miR-200c negative tumors” tumors grew ab initio under regular diet conditions. When tumors reached approximately 150 mm^3^ mice were randomized into 4 groups: regular diet and control treatment (−DOX −DXR), regular diet and DXR treatment with 5 mg/kg (−DOX +DXR, single DXR treatment), DOX diet and control treatment (+DOX −DXR, single hsa-miR-200c treatment) and DOX diet and DXR treatment (+DOX +DXR, double treatment). A cohort of 5 mice per group was used for animal study I and 10 mice per group were used for the xenograft mouse model II. Treatment day 0 correlates with the day of reaching 150 to 200 mm^3^ of tumor size. On days 0, 7, 14 and 21 each mouse was injected i.v. with the appropriate treatment. Where indicated mice diet was switched to the DOX diet on day 0 of the treatment. Mice were sacrificed by cervical dislocation once their tumor reached a tumor diameter bigger than 12 mm.

The control cell line MDA-MB 231 Tripz Ctrl was injected as described above into the left flank of 6-week-old female NMRI-nu mice. Mice were beforehand divided into two diet groups (*n* = 5), normal or DOX diet. Mice were sacrificed by cervical dislocation once their tumor reached the critical size of 800 mm^3^.

All animal experiments were performed according to the guidelines of German law for the protection of animal life and were approved by the district government of Upper Bavaria. Reference number: ROB-55_2-2532_Vet_02-19-20.

### 2.16. Clinical Impact Using Kaplan–Meier Plotter

The clinical impact of hsa-miR-200c (gene symbol: hsa-miR-200c) [45] and GSTM3 (gene symbol: 202554_s_at) [46] was analyzed using the Kaplan–Meier-Plotter (https://kmplot.com/analysis/) (accessed on 3 November 2022) [47]. Overall survival was depicted for the miRNA analysis, and for the mRNA analysis, the relapse-free survival is shown additionally restricted to the cohort of basal breast cancer subtype and neoadjuvant chemotherapy treatment. Further parameters used for the analysis can be found in the Supplementary Figure S9.

### 2.17. Software

The graphical abstract was created with BioRender.com (accessed on 10 October 2022).

### 2.18. Statistical Analysis

Statistical significance was calculated utilizing GraphPad Prism 7.04. To compare two samples an unpaired two-tailed Student’s *t*-test was used, and to compare more than two samples the one-way ANOVA with Tukey’s Multiple Comparison Test or 2 way ANOVA with Šídák’s or Tukey’s multiple comparison test was applied. * *p*  <  0.05, ** *p*  <  0.01, *** *p*  <  0.001, **** *p* < 0.0001. Values are displayed as mean with SD.

## 3. Results

### 3.1. Proteomic Analysis of a hsa-miR-200c Knockout upon Doxorubicin Treatment Reveals a Higher Abundance of the Glutathione Pathway

**Figure 1:**
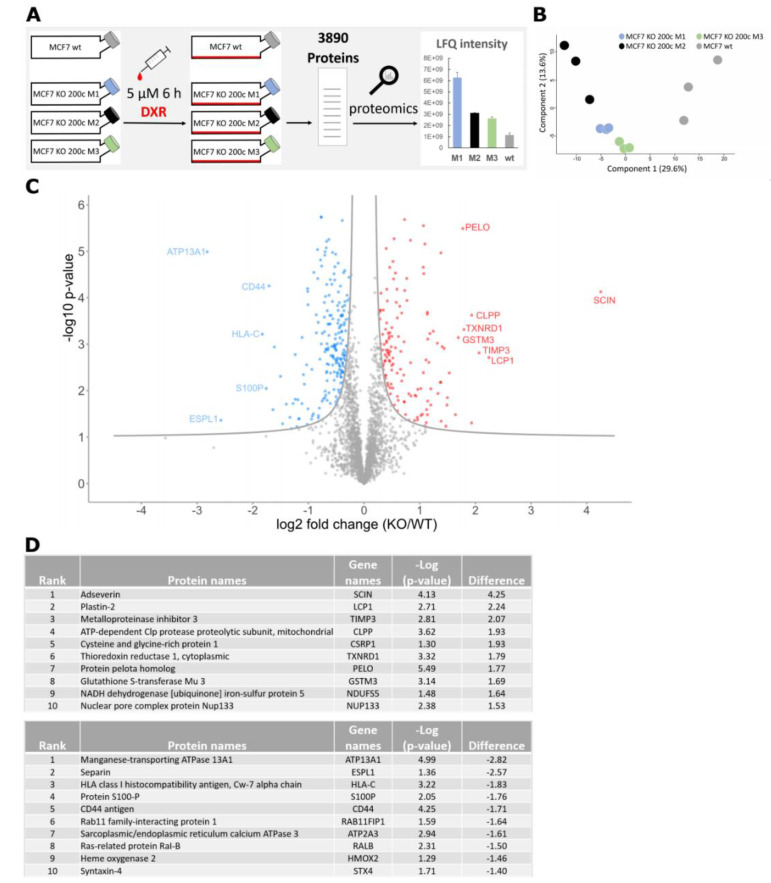
A proteomic analysis revealed novel hsa-miR-200c targets and altered signaling pathways in the field of detoxification from chemotherapy. (**A**) Experimental design of the proteomic approach of three monoclonal hsa-miR-200c knockout (KO) cell lines (M1: blue, M2: black and M3: green) vs. the parental MCF7 wt cells (gray) upon 6 h of 5 μM doxorubicin (DXR) treatment (*n* = 3). (**B**) Principal component analysis (PCA) of the hsa-miR-200c KO clones and MCF7 wt proteome profiles. Numbers in parentheses indicate the percentage of variation each component explains. (**C**) Volcano plot showing regulated proteins upon doxorubicin treatment in hsa-miR-200c positive MCF7 breast cancer cells vs. hsa-miR-200c KO cells. Significantly regulated proteins are indicated in red (upregulated) or blue (downregulated). (**D**) Top 10 list of proteins being significantly upregulated (upper table) or downregulated (bottom table) in DXR-treated MCF7 KO 200c cells compared to DXR-treated MCF7 wt. All genes are ranked for the biggest difference to MCF7 wt cells.

Several reports and reviews state that hsa-miR-200c is involved in drug resistance [3,8,12,18,28]. However, an in-depth analysis of the underlying mechanisms is lacking. Therefore, a proteome analysis was performed to evaluate the impact of altered hsa-miR-200c expression in the presence of chemotherapeutic treatment. Treated with doxorubicin (DXR), the wildtype (wt) epithelial luminal A breast cancer cell line MCF7 with high endogenous hsa-miR-200c expression was studied in comparison to three MCF7 hsa-miR-200c monoclonal knockout (KO) cell lines (M1, M2 and M3) [21]. A total number of 3890 proteins was identified in this approach (Figure 1A). The principal component analysis (PCA) of protein profiles depicted a clear separation indicating prominent differences between the KO and the wt cell lines when treated with DXR (Figure 1B). To identify differentially expressed proteins among the cell lines, a modified *t*-test (FDR < 0.05) was conducted, and the results were visualized in a volcano plot (Figure 1C). Here, 340 proteins were ascertained as significantly up- or downregulated in the KO cell lines compared to the unmodified parental MCF7 cell line. The top 10 most altered proteins are presented in Figure 1D. The majority of proteins, listed in Figure 1D, are part of cancer-relevant pathways such as proliferation (e.g., STX4, SCIN, TXNRD1), apoptosis (e.g., ESPL1, CD44, ATP2 A3, RALB, SCIN, TIMP3) and migration (e.g., S100 P, CD44, STX4, LCP1). Strikingly, glutathione S-transferase mu 3 (GSTM3), a protein that is involved in the detoxification of xenobiotics, such as doxorubicin, is highly upregulated in the hsa-miR-200c KO cell lines. Glutathione S-transferases (GSTs) convert xenobiotics to less toxic derivates and help to excrete them from the cells by conjugating glutathione to the drugs [11] and therefore play a crucial role in drug resistance of tumors [15]. To further identify affected pathways, a gene set enrichment analysis (GSEA) was performed. Here, overexpression of the Gene Ontology (GO) term “glutathione metabolic process” was detected. Besides GSTM3, three other altered glutathione S-transferases were identified, namely GSTK1, GSTZ1 and MGST1 (Figure S1A) and depicted in a heat map (Figure S1B). Additionally, unsupervised hierarchical clustering was accomplished on the proteins included in the GO term. (Figure S1C). Summarizing the performed proteomic analysis, evidence is shown that MCF7 KO cells, lacking hsa-miR-200c expression, upregulate a number of resistance-relevant proteins, amongst them several of the glutathione pathway, which are important for the detoxification of drugs.

### 3.2. Glutathione S-Transferase mu 3 Is a Novel Target of hsa-miR-200c-3p

**Figure 2:**
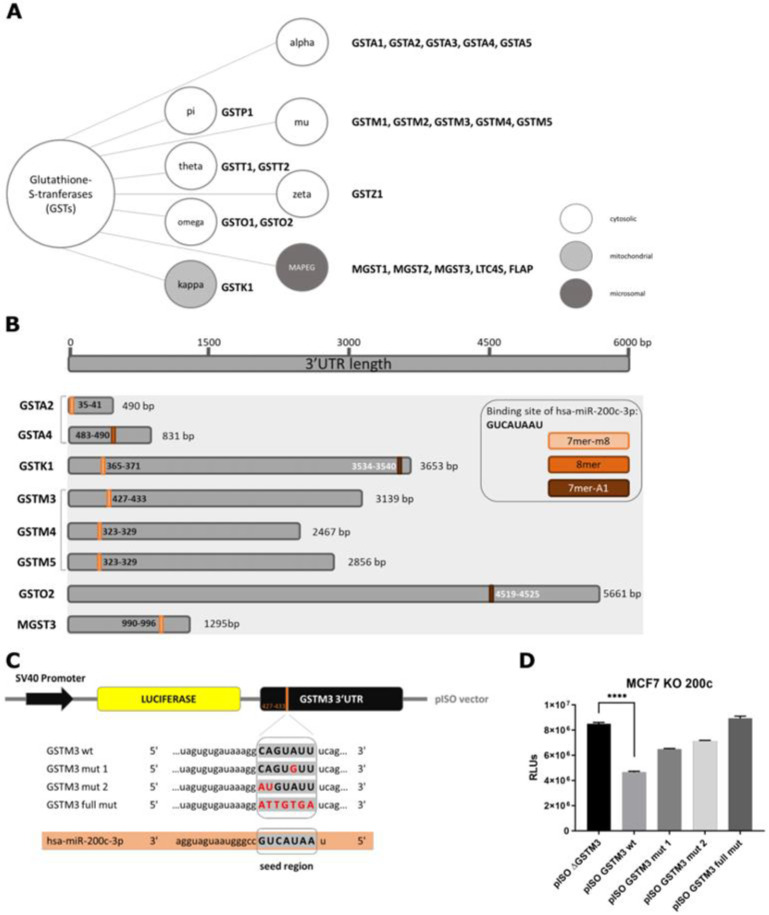
Validation of hsa-miR-200c-3p target site in the 3′UTR of glutathione S-transferases. (**A**) Glutathione S-transferases can be classified into 8 different families. (**B**) The 3′UTR regions and their length in base pairs (bps) are indicated for the glutathione S-transferases which show a target site for hsa-miR-200c. The orange/brown-colored bars display the localization of the hsa-miR-200c target site. (**C)** Schematic layout of the luciferase reporter plasmid. The unmodified (wt), as well as three different mutations (indicated with red letters) of the GSTM3 3′UTR, are shown in comparison to the seed region of hsa-miR-200c. (**D**) Luciferase assay of the different GSTM3 3′UTR constructs. Hsa-miR-200c and the plasmids were co-transfected into MCF7 KO 200c cells. One representative diagram out of three is displayed. A two-tailed Student’s *t*-test for pISO ∆GSTM3 and pISO GSTM3 wt was performed. **** *p* < 0.0001. Values are displayed as mean with SD.

Next, glutathione S-transferases as potential targets of hsa-miR-200c-3p were investigated in more detail. The family of GSTs can be classified into eight subgroups, consisting of one to five members (Figure 2A). Potential hsa-miR-200c-3 p target sites were found in silico throughout all GST families. Eight transferases show either a 7mer-m8, 8mer or a 7mer-A1 predicted binding site for hsa-miR-200c in their 3′UTR region (Figure 2B and Figure S2A). To analyze whether the identified sites are crucial for the regulation by hsa-miR-200c, the GSTM3 3′UTR was chosen as an exemplary target sequence and validated by a luciferase reporter assay. Part of the wt 3′UTR of GSTM3 was cloned downstream of a luciferase sequence and transfected into the MCF7 KO 200c cell line (Figure 2C). Synthetic hsa-miR-200c was co-transfected and thus a reduction in the relative light units (RLUs) was detected. Subsequently, the impact of sequence mutations of the hsa-miR-200c target site of the GSTM3 3′UTR was examined. The more the sequence of the target site was modified the higher was the ability to reconstitute the luminescence signal towards the initial level (Figure 2D). Further, a scrambled control siRNA was co-transfected with different GSTM3 3′UTR plasmids (Figure S2D) confirming that the 3′UTR of glutathione S-transferase mu 3 is a direct target of hsa-miR-200c. 

### 3.3. Hsa-miR-200c Controls the Expression of Additional Glutathione S-Transferases

**Figure 3:**
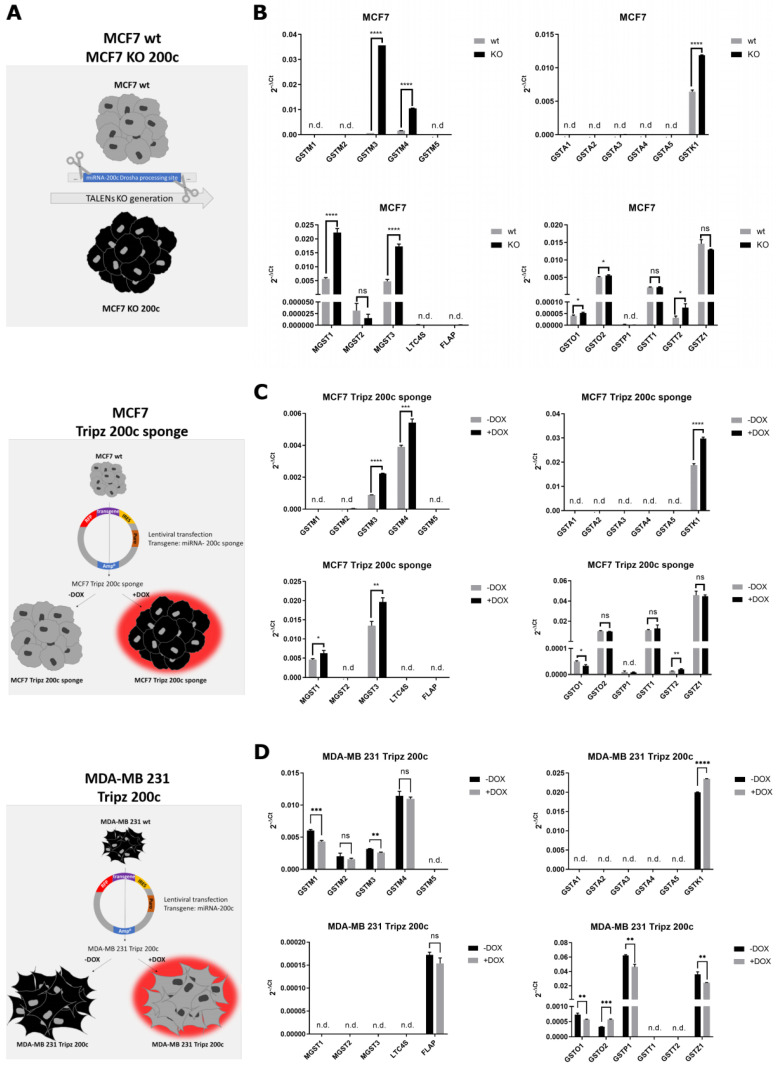
Expression of glutathione S-transferases (GSTs) in different hsa-miR-200c expression systems. (**A**) Overview of the generation of cell systems. Corresponding qRT-PCR analysis of all glutathione S-transferases in the (**B**) MCF7 wildtype and MCF7 KO 200c, (**C**) MCF7 Tripz 200c sponge and (**D**) MDA-MB 231 Tripz 200c cell line. Both Tripz construct systems were induced (or not) with 5 μg/mL doxycycline (DOX) for 72 h. An unpaired two-tailed Student’s *t*-test was performed. * *p* < 0.05, ** *p* < 0.01, *** *p* < 0.001, **** *p* < 0.0001. Values are displayed as mean with SD. ns = not significant, n.d. = not detected.

For a comprehensive analysis of the expression changes of all GSTs mediated by hsa-miR-200c, three different model cell lines were utilized. Besides the previously generated hsa-miR-200c KO cell line [21], a doxycycline-inducible MCF7 sponge cell line was generated (Appendix A) for the current study to scavenge the endogenous mature miRNA. Furthermore, a third cellular system, MDA-MB 231 Tripz 200c, was generated, in which the hsa-miR-200c and the red fluorescent protein (RFP) expression can be induced simultaneously in an hsa-miR-200c-null background (Figure 3A) [37]. Analyses of the expression levels and kinetics of the respective constructs and cell lines can be found in the Supplementary Figures S3A–C and Appendix B. Deactivating hsa-miR-200c in our two MCF7 cell systems led to the consistent upregulation of seven glutathione S-transferases (GSTM3, GSTM4, GSTK1, MGST1, MGST3, GSTO2, GSTT2) compared to the wt and uninduced cells, respectively (Figure 3B,C, Supplementary Figure S2B and Table S1). When hsa-miR-200c was overexpressed in the doxycycline-induced MDA-MB 231 Tripz 200c cell line, GSTM1, GSTM3, GSTO1, GSTP1 and GSTZ1 were downregulated (Figure 3D, Supplementary Figure S2B and Table S1). Comparing the eight GSTs harboring an in silico identified target site to the GSTs identified with quantitative RT-PCR (Table A1) revealed three GSTs resembling the expected changes in expression pattern (GSTM3, GSTM4, MGST3) consistent in all cell systems. Three GSTs were not at all expressed in the investigated cell lines. GSTK1 and GSTO2 show, as expected, increased expression upon hsa-miR-200c downregulation in MCF7 cells but also enhanced expression upon hsa-miR-200c induction in the cancer cell line MDA-MB 231. Despite no obvious target sites for hsa-miR-200c, both MGST1 and GSTT2 seemed to be regulated by hsa-miR-200c in MCF7 cells (Figure S2B). As enlightened in the luciferase reporter assay (Figure 2D), point mutations in the hsa-miR-200c binding site may still lead to the degradation of the targeted mRNA. Therefore, the 3′UTR sequences of these GSTs were re-analyzed and potential binding sites of hsa-miR-200c were found in silico (Figure S2C).

### 3.4. GSTM3 as Target of the hsa-miR-200c Is Differentially Expressed upon Chemotherapeutic Treatment

**Figure 4:**
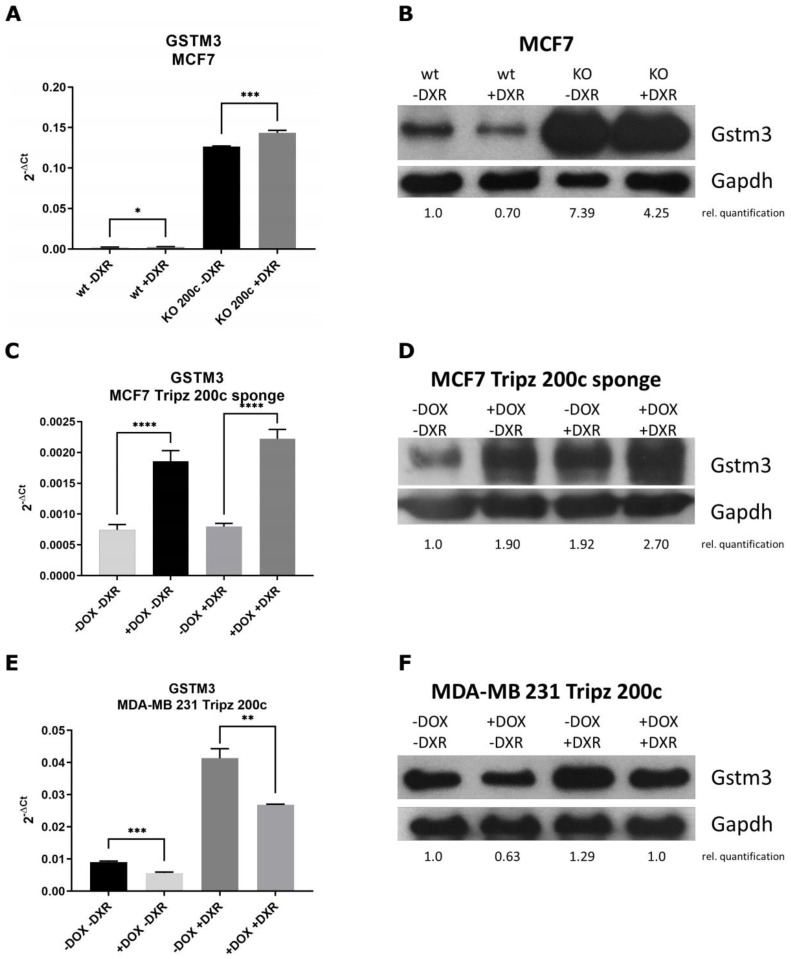
qRT-PCR and Western blot analysis of GSTM3 upon chemotherapeutic treatment. (**A,B**) MCF7 wt vs. MCF7 KO 200c cell line treated with 5 μM DXR for 6 h. (**C,D**) MCF7 Tripz 200c sponge cell line with or without DOX induction for 72 hours and subsequent DXR treatment with 0.1 μM for 24 h. (**E,F**) MDA-MB 231 Tripz 200c cells with or without DOX induction (72 h) and subsequent DXR treatment with 0.6 μM for 24 h. For Western blot quantification, contrast ratios were analyzed. An unpaired two-tailed Student’s *t*-test was performed for qRT-PCR statistics. One representative replicate out of three is shown. * *p* < 0.05, ** *p* < 0.01, *** *p* < 0.001, **** *p* < 0.0001. Values are displayed as mean with SD.

Further studies were carried out with GSTM3 only, as its hsa-miR-200c binding site was previously validated and because GSTM3 showed the most prominent expression change in the GST screen. We investigated the direct effect of doxorubicin and hsa-miR-200c on GSTM3 expression on mRNA (Figure 4A,C,E) and protein levels (Figure 4B,D,F and Supplementary Figure S10) in all three cell systems mentioned before. Generally, DXR treatment enhanced GSTM3 expression in all examined cell lines. The KO of hsa-miR-200c in MCF7 and the MCF7 sponge construct enhanced GSTM3 expression which was even elevated when cells were treated with DXR. On the contrary, hsa-miR-200c induction in MDA-MB 231 Tripz 200c cells led to a decreased expression of GSTM3. Thus we show, that when hsa-miR-200c expression is lost in cancer cells, GSTs will become upregulated. As a consequence, the increased number of GST enzymes promotes the binding of glutathione (GSH) to the drugs when patients’ tumors are treated with chemotherapy. Thereby, tumor cells become more resistant to chemotherapeutics as the drug is more rapidly inactivated and excreted. Hence, less accumulation of the chemotherapeutic drug in the cells will take place. 

### 3.5. Hsa-miR-200c Influences the GSH Pool and Mediates Drug Resistance In Vitro

**Figure 5:**
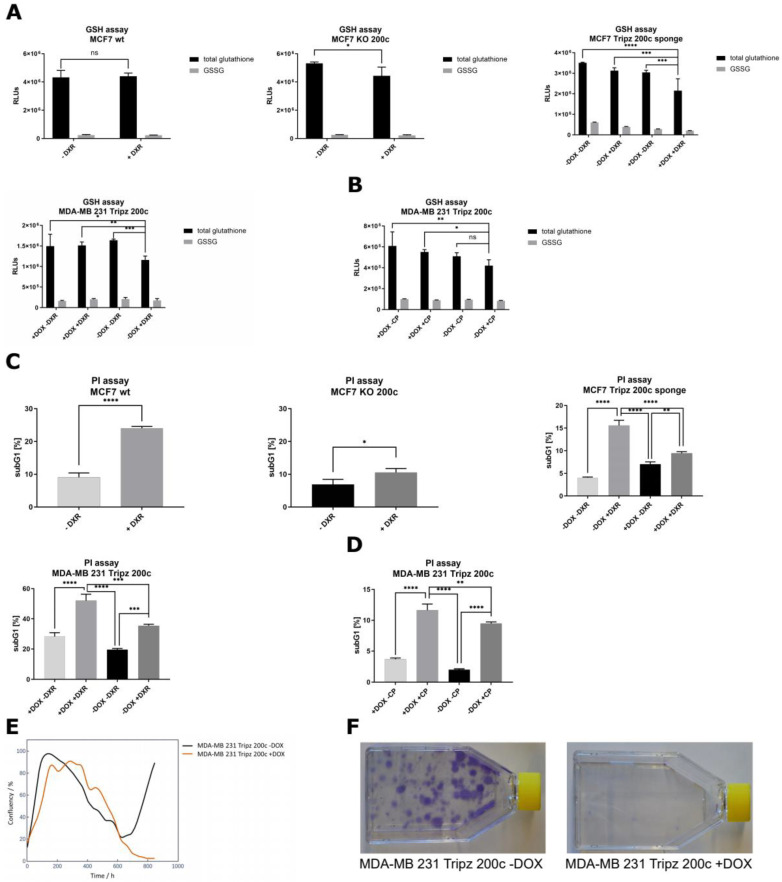
Effect of hsa-miR-200c expression on the glutathione (GSH) pathway and cell death. In vitro long-term effect of hsa-miR-200c expression. (**A**,**B**) GSH/GSSG assay in MCF7 wt vs. KO 200c, MCF7 Tripz 200c sponge and MDA-MB 231 Tripz 200c (doxorubicin left, cisplatin right). Total GSH and GSSG were measured after 24 h of DXR or CP treatment (0.1 μM DXR for MCF7 and 0.6 μM DXR and 50 μM CP for MDA-MB 231 cells). Tripz-constructs were pre-incubated with 5 μg/mL doxycycline or not for 72 h before chemotherapeutic treatment. (**C**,**D**) PI assay analysis using propidium iodide (PI) and measurement of subG1 population in MCF7 wt vs. KO 200c, MCF7 Tripz 200c sponge and MDA-MB 231 Tripz 200c. Either an unpaired two-tailed Student’s t-test, an ordinary one-way ANOVA with Tukey’s multiple comparison test or a 2 way ANOVA with Šídák’s or Tukey’s multiple comparison test was performed for statistics. One representative replicate out of three is shown. * *p* < 0.05, ** *p* < 0.01, *** *p* < 0.001, **** *p* < 0.0001. Values are displayed as mean with SD. (**E**) Confluency monitoring and corresponding (**F**) colony formation of MDA-MB 231 Tripz 200c cells induced (orange lines) or not (black lines) with DOX (every 48 to 72 h) and single treatment with 0.1 μM DXR at 80% of confluency.

To verify this hypothesis, different physiological assays were performed. A GSH/GSSG-Glo assay was carried out, as GSTs reduce the pool of GSH present in a cell by conjugating it to xenobiotics. Consistent in all three cell systems, the number of total glutathione is reduced when hsa-miR-200c is absent and the cells were stressed with DXR at the same time (Figure 5A). A second chemotherapeutic drug, i.e., cisplatin (CP), was utilized to show general validity. Similar to the treatment with DXR, the GSH/GSSG assay revealed a decrease in the total glutathione amount when hsa-miR-200c was absent and cells were treated with CP (Figure 5B).

The analysis of the subG1 population, representing the rate of cell death in general, disclosed that the highest proportion of apoptotic cells can be observed when cells express hsa-miR-200c and are treated with DXR simultaneously (Figure 5C). Similar results were obtained in all cell systems. Likewise, when cells were treated with CP, the highest subG1 levels, and therefore the most abundant rate of cell death was observed with high hsa-miR-200c expression levels (Figure 5D). To expand these findings from breast cancer to other tumor types, the A549 Tripz 200c lung cancer cell line and the T24 Tripz 200c bladder cancer cell line were generated. Upon hsa-miR-200c induction, chemosensitivity to DXR and CP was increased (Figure S4A–D). These data demonstrate on the one hand that tumor cells expressing hsa-miR-200c are more sensitive to chemotherapeutic treatment. On the other hand, by losing the hsa-miR-200c expression, tumor cells can acquire drug resistance.

To analyze the long-term effects of hsa-miR-200c expression, where drug resistance is even more evident, MDA-MB 231 Tripz 200c cells, whether induced with doxycycline (DOX) or not, were treated once with DXR and the cell confluency was monitored for six weeks. This live cell imaging can be retraced in the videos of both cell lines (Supplementary Videos S1 and S2). In line with the literature, we observed a retarded cell proliferation in hsa-miR-200c expressing cells, which is depicted by an increased doubling time and a flatter proliferation curve (Figure S5, Proliferation analysis method can be found in Appendix C). The one-time chemotherapeutic treatment led to the expected decrease in confluency, however, in hsa-miR-200c expressing cells this effect was significantly stronger. While in the experimental setting without hsa-miR-200c expression, different resistant clones started to regrow, no viable cells were detected when hsa-miR-200c expression had been induced (Figure 5E). This effect can also be illustrated by a colony formation assay where crystal violet staining represents regrown clones only in hsa-miR-200c negative cells (Figure 5F). 

### 3.6. Xenograft Mouse Models Present Drug Resistance In Vivo upon Modulation of hsa-miR-200c Expression

**Figure 6:**
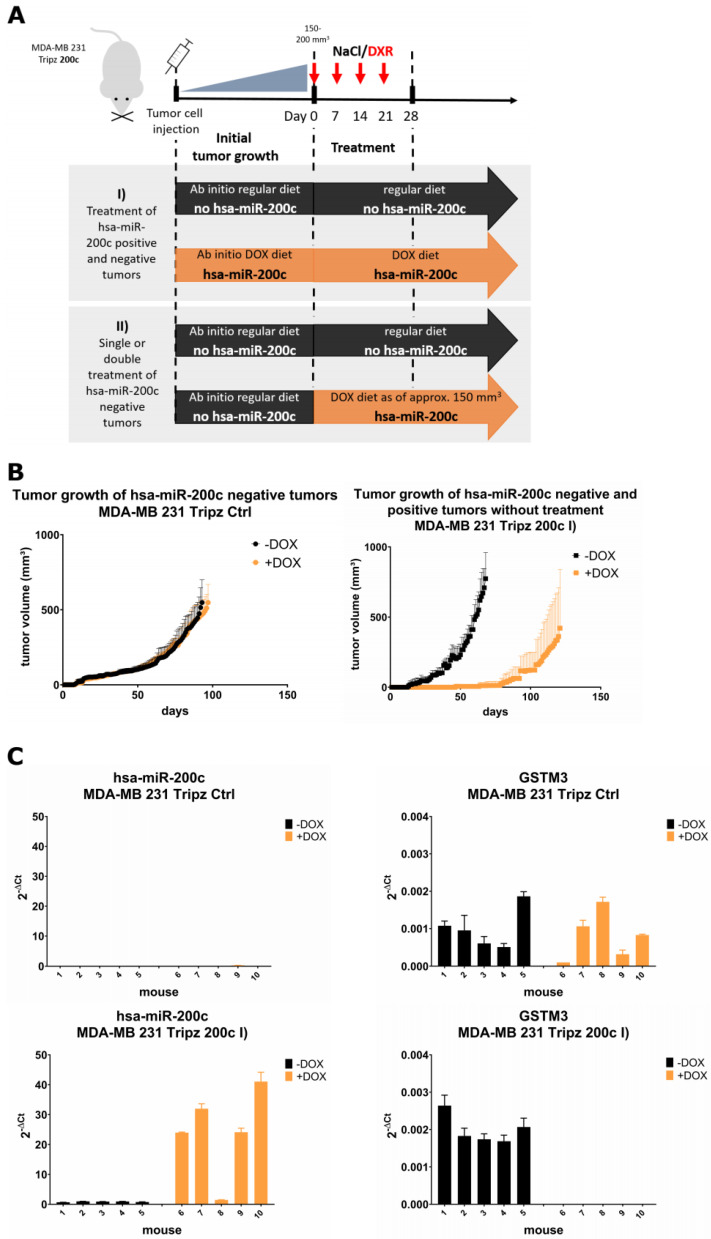

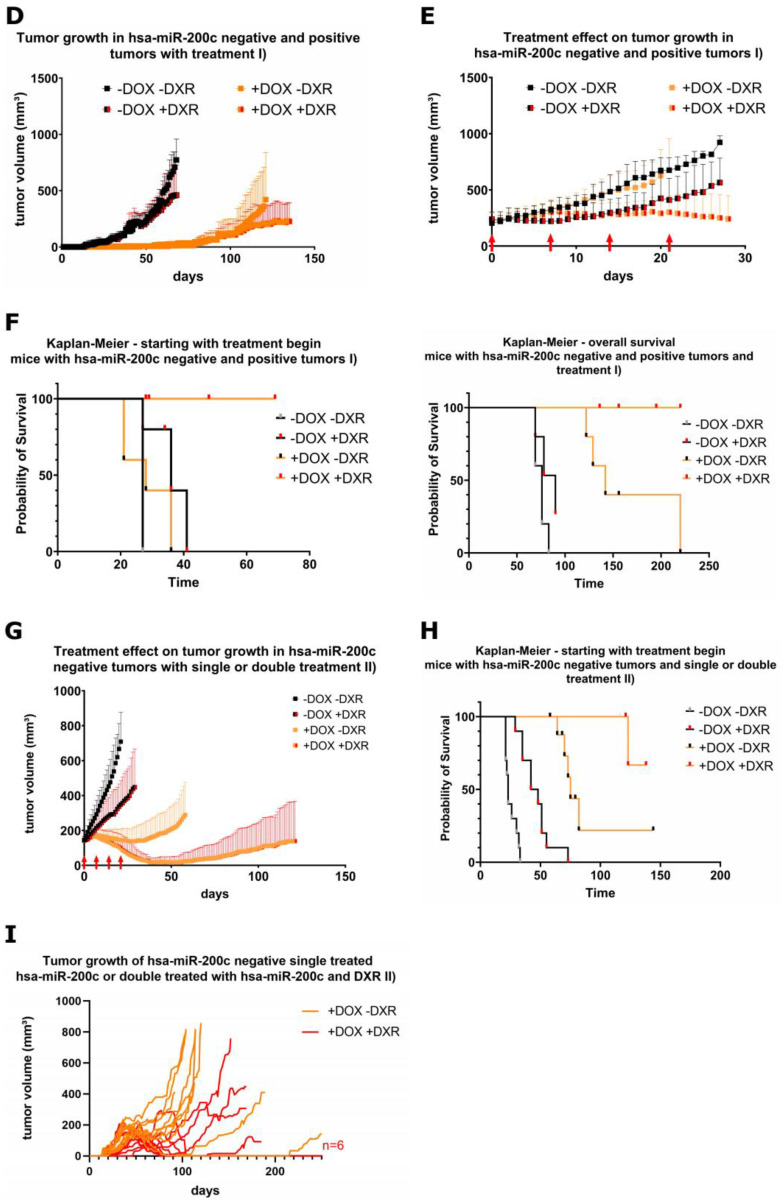
Long-term effect of hsa-miR-200c expression in vivo on tumor growth and resistance. (**A**) Two xenograft mouse models to investigate tumor growth: (I) Scheme for the analysis of hsa-miR-200c positive and negative tumors and additional chemotherapeutic treatment. (II) Xenograft mouse model of MDA-MB 231 Tripz 200c tumors to examine single doxorubicin (DXR) or hsa-miR-200c treatment and their combination. (**B**) Tumor growth of MDA-MB 231 Tripz Ctrl (control sequence expressing tumors) vs. MDA-MB 231 Tripz 200c (*n* = 5 per group) under regular (black) or DOX diet (orange). (**C**) Corresponding molecular analysis of the control (top) and hsa-miR-200c (bottom) inducible xenograft mouse model. Quantitative RT-PCR analysis of the expression levels of GSTM3 and hsa-miR-200c. DOX induction is indicated in orange. (**D**) Evaluation of the development of hsa-miR-200c positive (*n* = 5, orange curves) and negative tumors (*n* = 5, black curves) and their treatment with DXR (red-filled squares) based on tumor growth. (**E**) Tumor growth analysis normalized to treatment begin (set as day 0) in hsa-miR-200c positive and negative tumors with or without DXR treatment. Red arrows indicate treatment days (days 0, 7, 14 and 21). (**F**) Kaplan–Meier analysis of mice with hsa-miR-200c positive or negative tumors starting at treatment begin (left). Second Kaplan–Meier (right) displays the overall survival of mice with or without hsa-miR-200c expression and treatment. (**G**) The analysis of tumor growth of xenograft mouse model II). The development of tumor volume in hsa-miR-200c deficient mice upon only treatment with DXR or hsa-miR-200c or the combinatorial treatment (*n* = 10 per group). Red arrows indicate treatment days. (**H**) Kaplan–Meier survival analysis of mice with single or combinatorial treatment of hsa-miR-200c negative tumors (black curves). All tumor growth graphs terminate when the first animal of the group had to be euthanized. (**I**) Tumor growth curves displaying resistant, regrowing tumors of single mice from either the hsa-miR-200c treated (orange curves) or the hsa-miR-200c and DXR double-treated group (red curves). Red *n* = 6 presents the number of mice with complete regression in the double-treatment cohort at day 250 after tumor cell inoculation.

As hsa-miR-200c positive and negative tumors exist in the clinics, we investigated, on the one hand, the growth of these tumors, and on the other hand, tested our hypothesis of drug resistance by loss of hsa-miR-200c, in a xenograft mouse model. Therefore, we utilized the inducible MDA-MB 231 Tripz 200c cell model and an inducible MDA-MB 231 Tripz Ctrl cell line, expressing a control RNA sequence upon DOX administration. Respectively, one group of mice was fed with DOX feed continuously from the day of tumor cell injection on to induce the expression of the hsa-miR-200c transgene in all tumor cells (Figure 6A,I) “Treatment of hsa-miR-200c positive and negative tumors”). No difference in tumor growth was detected in both control cell lines (Figure 6B) which indicates that the steady DOX diet does not affect tumor growth. As expected, the hsa-miR-200c expressing group showed delayed and strongly decelerated tumor growth compared to the control group (Figure 6B). After reaching a tumor size of 150–200 mm^3^ two additional groups of mice were introduced to the study. From each diet group (with or without DOX feed, *n* = 10) mice were either treated i.v. with 5 mg/kg DXR (*n* = 5) or with NaCl-solution (*n* = 5). After mice had been sacrificed, the expression of hsa-miR-200c and GSTM3 was analyzed. In the control groups (MDA-MB 231 Tripz Ctrl), where hsa-miR-200c cannot be expressed, GSTM3 was constantly expressed, irrespective of the DOX diet. In mice where hsa-miR-200c was induced in the tumors by DOX (MDA-MB 231 Tripz 200c), cells expressed hsa-miR-200c to a high level and, in line with the in vitro results, did not express GSTM3 (Figure 6C). Sections of hsa-miR-200c positive tumors did not show histological differences compared to hsa-miR-200c negative tumors, and no loss in body weight could be observed in any groups independent of the cell line, the induction or treatment (Figure S6A–D; H&E staining method is depicted in Appendix D).

When analyzing the effect of chemotherapy on tumor growth and survival, reduced tumor size was measurable in both groups treated with the chemotherapeutic drug (Figure 6D). Of note, mice only harboring hsa-miR-200c expressing tumor cells, which did not receive the anti-cancer treatment, revealed a strong reduction in tumor growth. However, these results are strongly influenced by the long-term effect of decelerated proliferation in hsa-miR-200c positive cells. To level these effects, we normalized the tumor growth of all mice to the starting day of chemotherapeutic treatment. Here, a cytostatic effect of the chemotherapeutic treatment was measurable whereas a cytotoxic effect was only observed in hsa-miR-200c expressing and DXR-treated tumors (Figure 6E). Increasing tumor volumes were measured in all groups until the euthanasia of the mice except for the DXR-treated group of hsa-miR-200c positive tumors. The latter group comprised of mice with slowly growing tumors (*n* = 1), with static tumors (*n* = 3) and with completely reduced tumors (*n* = 1) till euthanasia of mice, reflecting the cytostatic effect of doxorubicin in hsa-miR-200c positive tumors. Thus, hsa-miR-200c-positive tumors are more chemosensitive than tumors without hsa-miR-200c expression. When evaluating the survival rate from the start of the treatment, most mice had to be sacrificed within 41 days (Figure 6F left). Only in the case of hsa-miR-200c positive tumors and DXR treatment a prolonged survival was observed, and mice had to be sacrificed due to different animal health care reasons. When taking also the days before treatment into account, the Kaplan–Meier analysis indicates that hsa-miR-200c negative tumors were growing faster and reached the critical tumor size earlier (Figure 6B). Consequently, the treatment of these mice had to start approximately 60 days prior to the treatment of the first mice with hsa-miR-200c positive tumors. Mice without hsa-miR-200c expression showed the worst overall survival compared to hsa-miR-200c expressing mice (Figure 6F right). Concluding from these data, the expression of hsa-miR-200c in cancer cells shows a dramatic delay of tumor growth onset and an increased sensitivity to chemotherapy. 

In a second experiment (Figure 6A, II) “Single or double treatment of hsa-miR-200c negative tumors”) mice without hsa-miR-200c induction were randomized after the tumors reached a volume of 150–200 mm^3^. To investigate whether hsa-miR-200c could sensitize tumor cells to DXR treatment, groups (*n* = 10) of mice were formed that were either only DXR treated, or only hsa-miR-200c induced with DOX, and a cohort of mice with a combination of both was formed. We observed a clear beneficial effect of the double (+ DOX + DXR) treatment in tumor growth (Figure 6G). Survival analysis revealed that mice without hsa-miR-200c expression, irrespective of DXR treatment, had to be euthanized within the first 73 days after the beginning of the treatment, whereas mice with the induction of hsa-miR-200c during the tumor growth were partially still alive at the end of the study. Drug treatment showed a beneficial survival rate for both diet groups but an impressive increase when combined with hsa-miR-200c expression (Figure 6H). A detailed analysis of the tumor growth of single mice in the cohort of hsa-miR-200c treatment versus the double treatment group revealed different tumor growth after the initial response to both treatments (Figure 6I). Mice, treated with DOX showed a tumor growth progression after a while. Only two mice of this group were observed with tumor regression followed by a recurrence of the tumor. In comparison, mice additionally being treated with doxorubicin showed delayed tumor progression (*n* = 3) or recurrence after a while (*n* = 1). The remaining animals from the double treatment group (*n* = 6) displayed no recurrence until this study was terminated 250 days after tumor cell injection.

**Figure 7:**
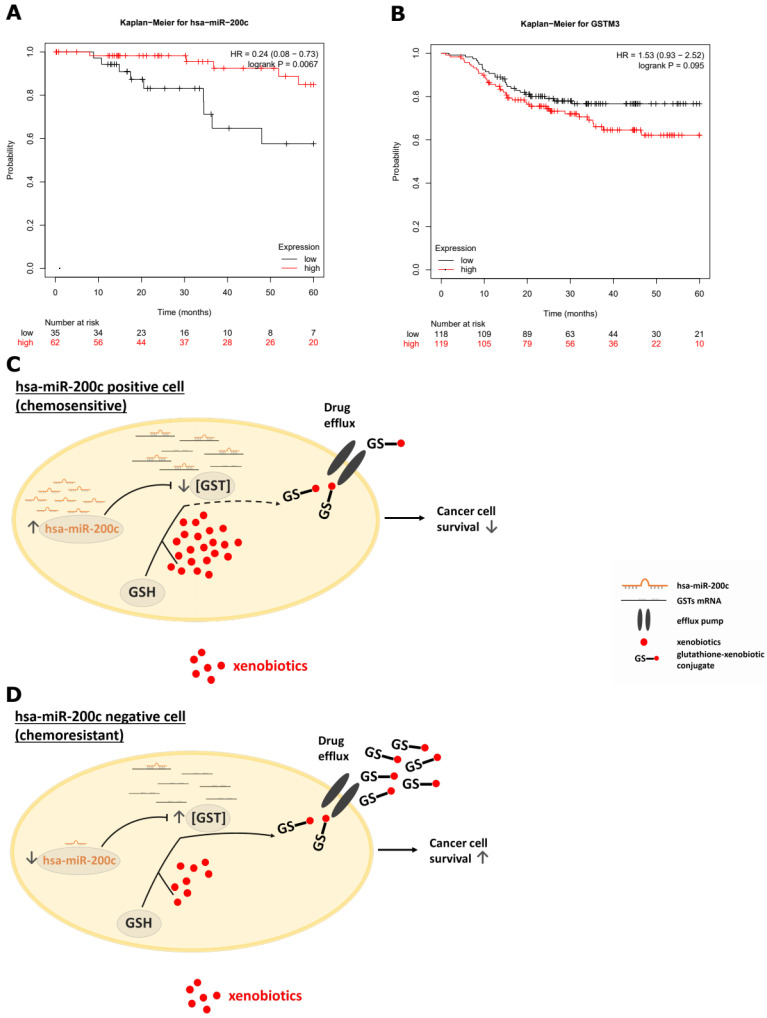
Analysis of clinical relevance. (**A**) Kaplan–Meier analysis for hsa-miR-200c expression in breast cancer patients. Overall survival is depicted. (**B**) Kaplan–Meier plot for the relapse-free survival of GSTM3 expressing patients. Neoadjuvant chemotherapy and basal subtype were used as cohort. (**C**) Graphical summary and hypothesized mechanism of action of hsa-miR-200c and its target GSTM3 in cancer cells. Potential detoxification pathway of a hsa-miR-200c positive cell upon treatment with xenobiotics such as doxorubicin. (**D**) Putative mechanism of resistance formation in hsa-miR-200c negative cells.

Whether the in vivo results are reflected in the clinics, an in silico study on breast cancer patients was performed. The Kaplan–Meier analysis revealed a significant increase (*p* = 0.0067) in the overall survival of patients with tumors displaying high hsa-miR-200c levels compared to patients with low hsa-miR-200c expression (Figure 7A and Supplementary Figure S9). To analyze the hsa-miR-200c target GSTM3, a patient cohort with neoadjuvant chemotherapy treatment and displaying a basal subtype was plotted. Comparing the 5-year relapse-free survival, a reduction in the probability of survival in the GSTM3 overexpressing cohort could be seen (Figure 7B and Supplementary Figure S9). 

Taking all results into account, we establish the hypothesis that hsa-miR-200c positive tumors suppress GSTs’ expression, which is beneficial for a successful chemotherapy by increasing the overall survival of cancer patients. In this case, xenobiotics remain longer in the tumor cells and cause greater damage, which results in enhanced tumor cell death. On the contrary, loss of hsa-miR-200c leads to increased expression of glutathione S-transferases resulting in better drug export, drug-resistant tumors and eventually reduced overall survival of the patients (Figure 7C,D).

## 4. Discussion

The present study focuses on the influence of hsa-miR-200c on drug resistance. To make sure the findings have broad significance, we used three different cell systems regulating hsa-miR-200c expression in breast cancer in vitro. In the proteomic approach, a hsa-miR-200c knockout (MCF7 KO 200c) was utilized, because only targets are supposed to show up that are regulated under physiological conditions. In contrast, inducible overexpression of hsa-miR-200c can lead to the repression of less significant targets which contain target sites but would not be regulated under physiological conditions. However, this fact is beneficial in a therapeutic setting when wide spectra of highly expressed oncogenes need to be targeted. Nevertheless, an inducible overexpression system, based on an aggressive triple-negative breast cancer cell line (MDA-MB 231 Tripz 200c) was also used to validate our results. The KO of hsa-miR-200c hampers the processing of the pri-miRNA to pre-miRNA and thus the expression of both miRNA strands (3p and 5p) is prevented [48]. Targets of either the 3p or 5p strand are therefore included in the proteomics data. The MCF7 Tripz 200c sponge cell line was generated with the purpose to scavenge the mature miRNA strand. 

The proteome analysis identified, besides others, GSTM3 and the glutathione pathway to be differentially regulated. This pathway is part of the fundamental phase II detoxification mechanism in cells [9,10,11]. Elevated activity of the glutathione S-transferases is associated with a decrease in the intracellular concentration of chemotherapeutic drugs, thus contributing to drug resistance [9,13,49]. Many GSTs, e.g., GSTO1, GSTP1, GSTK1, GSTA4 and GSTM3, are reported to cause resistance in cancer [50,51,52]. A prominent GST in the resistance formation of breast cancer is GSTP1 [53,54], which was also observed to be regulated by hsa-miR-200c in the presented experiments. Enhanced detoxification effects arise in this case from either GSTP1 overexpression and increased drug efflux or from targeting the MAP kinase pathway [9,54]. Experiments in the present report revealed for the first time a large number of GSTs to be controlled by hsa-miR-200c, which was validated in the three hsa-miR-200c expression systems. The predicted hsa-miR-200c binding site in the 3′UTR of GSTM3 mRNA, which was the most differentially expressed GST, was validated. Five other GSTs displayed the same binding site, and similar sites (8mer and 7mer-A1) were found in two more GSTs. Examining the mRNA expression of all GSTs, the in silico results were partly confirmed. Additional regulation of GSTs was also observed, indicating that the sequence of the binding sites might not be strictly determined. This is in line with our binding site mutations where, despite two mutated base pairs, still a reduced luciferase signal was detected when hsa-miR-200c had been transfected. Interaction of miRNAs with other regions besides the 3′UTR, as, e.g., the 5′UTR, the coding sequence, and gene promoters, were also reported which might be the case in the regulation of several other differentially regulated GSTs [19]. 

Up to now, hsa-miR-200c has not been reported to regulate the expression of GSTs and is thus not considered a player in the detoxification via glutathione S-transferases. Phase II detoxification is facilitated by GSTs, which catalyze the conjugation of GSH to xenobiotics. Therefore, it was not surprising to observe reduced cellular GSH pools when the hsa-miR-200c function was disabled, i.e., GSTs became overexpressed, and cells were stressed by doxorubicin (DXR) and cisplatin (CP). Additionally, CP as well as DXR treatment of hsa-miR-200c-positive cells led to an increase in cell death evidenced by higher subG1 levels. Therefore, chemotherapeutics show less efficacy on hsa-miR-200c negative cells which consequently acquire drug resistance. We additionally investigated a potential direct effect of hsa-miR-200c on p-glycoprotein expression, i.e., ABCB1, which is known to also play a pivotal role in the development of drug resistance by enhancing the drug efflux through elevated expression [55]. We measured only very low mRNA expression of ABCB1 in our cell lines and thus did not detect a significant regulation by hsa-miR-200c (Figure S8, Table A2). Besides breast cancer cell lines, the in vitro studies were extended to additional types of cancer such as lung cancer (A549 cell line) and bladder cancer (T24 cell line). Both cell lines show endogenously a very low hsa-miR-200c expression level (Figure S7A) which is inducible upon DOX administration (Figure S7B). As in the breast cancer cell lines, introducing hsa-miR-200c led to sensitization towards the chemotherapeutic drugs DXR and CP. Similar findings were published with gemcitabine in pancreatic cancer cells [56] and in A549 cells, where the role of the miR-200bc/429 cluster on drug resistance was investigated [57]. Thus, hsa-miR-200c expression might play an important role in resistance formation in breast, lung and bladder cancer.

To transfer these results to in vivo studies, we conducted two mouse xenograft experiments. First, similar to the clinical situation of patients, where hsa-miR-200c positive and negative tumors exist, we started the induction of hsa-miR-200c expression in the cell line MDA-MB 231 Tripz 200c at the moment of tumor cell inoculation. Here, the late tumor growth onset and the reduced proliferation reflect our in vitro studies and are in line with previously published data [37,58,59,60,61,62,63]. This effect is very likely mediated by hsa-miR-200c, regulating a number of other proliferation-associated proteins, most important Kras [23,60]. In the study of Schilb et al., where hsa-miR-200c was delivered via nanoparticles to MDA-MB 231 cells, a similar effect was observed [64]. In our experiments, DXR treatment reduced tumor growth in both groups (hsa-miR-200c positive and negative tumor cells). However, mice with hsa-miR-200c positive tumors showed even more reduced tumor growth, in some cases even a complete halt. This additive effect in diminished tumor growth and extended survival in vivo can be exploited to reduce the dose of a chemotherapeutic drug. In the case of doxorubicin, severe side effects such as cardiotoxicity could be attenuated [65].

The most important question was to evaluate whether the presence of hsa-miR-200c has an effect on the efficacy of chemotherapeutic treatment. For this reason, tumors were grown without hsa-miR-200c expression and mice were randomized when tumors reached a size of 150–200 mm^3^. In the group obtaining the double treatment regime, i.e., hsa-miR-200c expression and DXR treatment, all mice showed a regression of tumor volume and in 6 out of 10 mice eventually a complete response until the end of his study (day 250 after tumor cell injection). The difference in the response to the treatment in both xenograft mouse models can be attributed to the fact that in one case tumors developed under a constant DOX diet and could therefore get accustomed to the expression of this miRNA. An adaption effect of these tumors towards hsa-miR-200c is also reflected by the similar growth rate of hsa-miR-200c positive and negative cells at the later tumor growth phase (Figure 6E). To our knowledge, up to now, only in vitro studies investigating the potential combination of hsa-miR-200c and drug treatment were performed [66]. Taken together, hsa-miR-200c expression is beneficial for an enhanced chemotherapeutic sensitivity of cancer cells, which is also reflected by our Kaplan–Meier plots analyzing the overall survival of breast cancer patients. The Kaplan–Meier curves represent the patients’ survival probabilities for hsa-miR-200c positive and negative breast cancer tumors. A loss of this miRNA is associated with a worse survival score (Figure 7A). Generally, this is possible through gene deletion, epigenetic mechanisms such as the methylation of DNA and histone modifications of the miRNA genes, altered expression of transcription factors, deregulation of the miRNA biogenesis and the expression of competitive endogenous RNAs (ceRNAs) [3]. In the case of hsa-miR-200c various mechanisms have been confirmed, such as the aberrant DNA methylation of CpG island in the promoter region of the miR-200c/141 cluster [67], binding of zinc finger E-box binding homeobox 1 (ZEB1) alone or together with the transcription factors CtBPs (C-terminal binding proteins) and BRG1 (Brahma-related gene-1) suppressing the expression of hsa-miR-200c [18], the basic helix-loop-helix transcription factor Achaete Scute-like 2 (Ascl2) as a potential transcriptional repressor [68] and expression of long non-coding RNAs (lncRNAs) such as TMPO-AS1 [69] and XIST [70] in the ceRNA network. Furthermore, the Kaplan–Meier plot reveals a survival benefit of breast cancer patients expressing hsa-miR-200c. This effect is potentially mediated by the hsa-miR-200c/GSTM3 pathway, resulting in a reduced phase II detoxification of tumor cells. Whether other upstream regulators of GSTM3 such as the lncRNA GAS5 [71] could also play a role in resistant cancer cells needs to be examined further. 

## 5. Conclusions

In conclusion, the present study highlights a superior effect of combined hsa-miR-200c expression with chemotherapeutic drug treatment both in vitro and in vivo. We uncovered a link between hsa-miR-200c expression and the glutathione metabolism leading to resistance formation of tumors. To resensitize tumors to chemotherapeutic drugs the reduction in the cellular phase II detoxification is mandatory. This was achieved by the induction of hsa-miR-200c expression and consequently a diminished expression of GSTs, e.g., GSTM3. Thus, it seems promising to establish hsa-miR-200c as a novel prognostic biomarker that can predict the success of chemotherapeutic treatment of cancer patients.Additionally, targeted hsa-miR-200c delivery into the tumor might act as direct nucleic acid therapeutic, extending the survival time of cancer patients by reconstituting chemosensitivity.

## Data Availability

All unique plasmids and all stable cell lines generated in this study are available from the corresponding author upon reasonable request. The data generated in this study are available on request from the corresponding author. The mass spectrometry proteomic data have been deposited to the ProteomeXchange Consortium (http://proteomecentral.proteomexchange.org, accessed on 14 October 2021) via the PRIDE partner repository [72] with the dataset identifier PXD029147.

## Supplementary Materials

The following supporting information can be downloaded at: https://www.mdpi.com/article/10.3390/cancers14225554/s1, Figure S1: Proteomic analysis of MCF7 KO 200c (M1, M2, M3) vs. MCF7 wt upon doxorubicin (DXR) treatment. Differential protein expression depending on the hsa-miR-200c expression.; Figure S2: In silico miRNA-mRNA seed-site interaction.; Figure S3: Characterization of hsa-miR-200c expression systems.; Figure S4: Effect of hsa-miR-200c on cell death in two additional cancer types in combination with chemotherapeutic treatment.; Figure S5: Proliferation curves of hsa-miR-200c positive or negative MDA-MB 231 cells.; Figure S6: Animal welfare monitoring of the different xenograft mouse models and histological analysis of hsa-miR-200c-positive and negative tumors.; Figure S7: hsa-miR-200c expression profile; Figure S8: hsa-miR-200c expression and the abundance of ABCB1.; Figure S9: Overview of the parameters entered into the Kaplan–Meier Plotter for the analysis of hsa-miR-200c and GSTM3 in breast cancer patients.; Figure S10: Uncropped blots of western blots presented in Figure 4B,D,F. Video S1: Live cell imaging of MDA-MB 231 Tripz 200c uninduced (-DOX) with doxycycline and additional doxorubicin treatment; Video S2: Live cell imaging of MDA-MB 231 Tripz 200c induced (+DOX) with doxycycline and additional doxorubicin treatment. Table S1: Raw Ct values of qRT-PCR analysis of all glutathione S-transferases in different hsa-miR-200c expression systems.

## Appendix A. Supplementary Material and Methods

Generation of stable MCF7 Tripz 200c sponge, A549 Tripz 200c and T24 Tripz 200c cell lines: To generate the inducible sponge, MCF7 wt cells were transduced with a 2nd generation lentiviral system comprised of the plasmids pCMV-dR8.2 dvpr (Addgene plasmid # 8455) and pCMV-VSV-G (Addgene plasmid # 8454). Both plasmids were a gift from Bob Weinberg [73]. The transfer plasmid is a modification of the doxycycline-inducible Tripz-Ctrl (ThermoFisher Scientific, #RHS4743) plasmid. The control sequence in the Tripz construct was substituted by the sponge sequence (GCAGAATGGGCATCACAAACCAATT) which consists of 12 repeats. After successful transduction, cells were selected with 5 µg/mL puromycin (Sigma-Aldrich, cat. no. P8833) for 48 h. Subsequently, a monoclonal MCF7 Tripz 200c sponge cell line was generated by performing single-cell dilution. The inducible Tripz 200c construct was stably transfected into A549 wt and T24 wt (purchased from ATCC) as previously described [37].

For the analysis of the ABCB1 gene, the TaqMan assay (ThermoFisher Scientific) with the TaqMan^®^ Fast Advanced Master Mix (ThermoFisher Scientific, cat. no. 4444557) was used and run on the LightCycler 480. Per sample, 5 µL of Master Mix was pre-mixed with 0.5 µL of the ABCB1 TaqMan assay. GAPDH was used as a housekeeper. Finally, 4.5 µL of a 1:10 dilution of cDNA was added to each well.

**Table A1:** Sequences of qRT-PCR primers and TaqMan qRT-PCR analysis.

	Left Primer	Right Primer	Probe
GSTA1	gagaactattgagaggaacaaagagc	tctcctggaggtttctctaagc	85
GSTA2	agaaacctccaggagactgcta	tctgccccgtatattggagt	53
GSTA3	ctgacattagcctggtggaac	tcagcagagggaagttggag	19
GSTA4	cttcctcttgtcctttgtcctc	tgctgccatgatagcttttc	58
GSTA5	tgcagaagatttggacaagttaag	ggttgtatttgctggcaatg	21
GSTP1	catctccctcatctacaccaacta	aggacctcatggatcagcag	62
GSTM1	aggacttcatctcccgcttt	cccagacagccatctttga	13
GSTM2	catgacactggggtactgga	tcctcgtagcttgagtctgtgt	68
GSTM3	ccaatggctggatgtgaaat	tccaggaggtagggcagat	85
GSTM4	tgacctctctgactgggaca	tctgaaggccagagaaccag	13
GSTM5	tggacgccttcctaaacttg	aaacaaaagacctcggaggaa	13
GSTT1	gtagccatcacggagctgat	gaagaggtcctcccccact	71
GSTT2	gctgtttcttgacctggtgtc	tcttgtgctgccctttgac	28
GSTZ1	cctgcagaacctgtctgtcc	ccacaagtgatggcgttct	55
GSTO1	gcacttttgagctaaggaggaa	caggggattcaggaagtaggt	12
GSTO2	gagatgtgggagagaatgcac	gaaggtggtgttctgatactcaag	8
GSTK1	tatttggctctgaccggatg	ggtatagggcccatccactt	62
MGST1	tcagcatccagttggctttt	aatgggtttaccccagttca	6
MGST2	gggtcaccagagtttgagaga	ccttgaagtgacgctgatga	85
MGST3	actggtgctgccagctttat	tttcagggtccgtgctgta	49
LTC4 S	accatgaaggacgaggtagc	tgcagggagaagtaggcttg	66
FLAP	catcagcgtggtccagaat	caagtgttccggtcctctg	52
GAPDH	tccactggcgtcttcacc	ggcagagatgatgaccctttt	45
hsa-miR-200c-3p	gcgtaatactgccgggtaat	PerfeCTa Universal PCR Primer	
hsa-miR-191	gcgcaacggaatcccaaaag	PerfeCTa Universal PCR Primer	

**Table A2:** TaqMan assays for qRT-PCR analyses with the LightCycler 480.

Proteins	Assay ID
ABCB1	Hs00184500_m1
GAPDH	Hs02758991_g1

## Appendix B. Quantification of RFP Expression in the Inducible Cell Systems

Cells were seeded in triplicates in 96 well plates and subsequently induced with 5 µg/mL DOX per well. Red fluorescence protein (RFP) expression was measured using FACS analysis. The evaluation was performed using FlowJo 7.6.5.

## Appendix C. Proliferation Analysis Using the PHIO Cellwatcher

Cells were seeded and induced with 5 µg/mL DOX where indicated. Every 48 to 72 h new DOX was added to the cells to compensate for depletion. The proliferation was determined as the doubling time value. The proliferation curve was analyzed by taking the confluency values between 30% and 60% into account. Doubling time was calculated using the equation of the best fit line and calculating the interval between 30% and 60% confluency.

## Appendix D. H&E Staining of In Vivo Tumors

H&E staining was performed as described earlier [74].
