# Supplementary material for: Combating Drug Resistance by Exploiting miRNA-200c-Controlled Phase II Detoxification
Source: Cancers (Basel). 2022 Nov 11;14(22):5554. doi: 10.3390/cancers14225554 (PMC9688189; doi:10.3390/cancers14225554)
Supplement: Supplementary file 1 [file cancers-14-05554-s001.zip › SI/Supplementary Information.pdf]

Figure S1

A

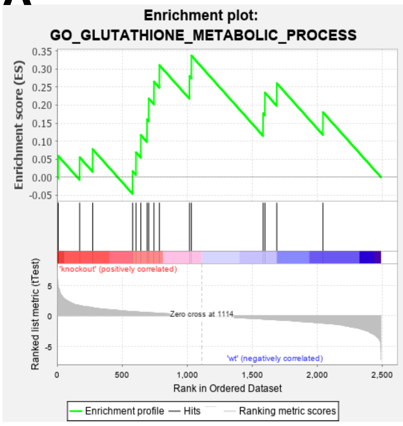

Gene	Rank in gene list	Rank metric score	Running ES	Core enrichment	P-value	Adj. p-value	Fold change
GSTM3	9	4.81225	0.0588622	Yes	0.0007	0.0058	3.2
G6PD	174	1.65421	0.0550728	Yes	0.0001	0.0013	1.2
GSTK1	274	1.29608	0.0775566	Yes	0.1477	0.3940	1.2
PARK7	581	0.647684	0.0163703	Yes	0.0544	0.2903	1.1
ALDH5A1	607	0.614908	0.0687652	Yes	0.1996	0.4144	1.1
GSTZ1	644	0.557833	0.116714	Yes	0.3008	0.4144	1.1
SOD1	692	0.491151	0.160216	Yes	0.6070	0.6937	1.1
GGCT	705	0.478132	0.217866	Yes	0.3367	0.4144	1.1
CNDP2	745	0.433922	0.264602	Yes	0.2601	0.4144	1.1
GSR	787	0.37562	0.31053	Yes	0.3117	0.4144	1.1
GLO1	1018	0.108919	0.280063	Yes	0.7180	0.7180	1.0
GSS	1033	0.0955065	0.336904	Yes	0.7036	0.7180	1.1
GCLC	1584	-0.558671	0.177092	No	0.2598	0.4144	0.9
IDH1	1598	-0.577975	0.234337	No	0.1296	0.3940	0.9
HAGH	1689	-0.692166	0.260459	No	0.2727	0.4144	0.9
MGST1	2044	-1.23087	0.179871	No	0.0889	0.3556	0.7

B

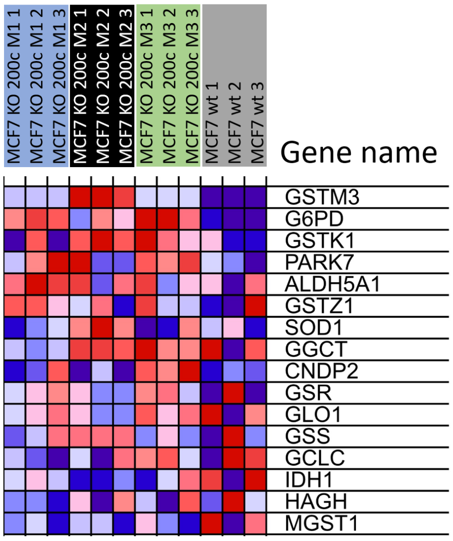

C

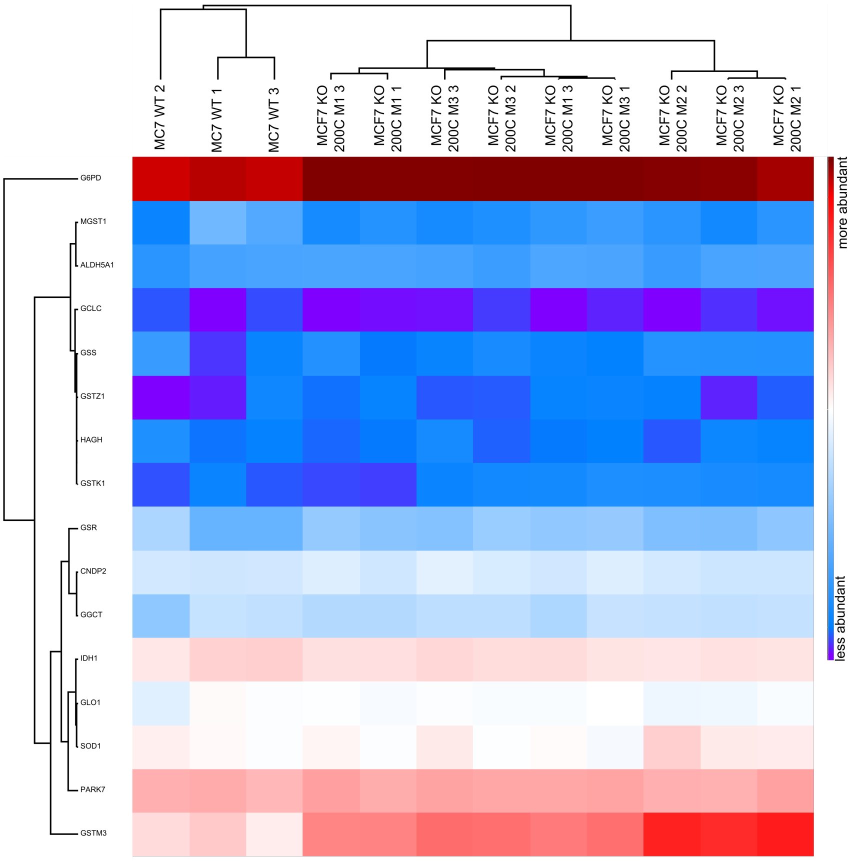

Figure S1. Proteomic analysis of MCF7 KO 200c (M1, M2, M3) vs. MCF7 wt upon doxorubicin (DXR) treatment. Differential protein expression depending on the hsa-miR-200c expression. (a) Gene set enrichment plot of the glutathione metabolic process pathway (left) and table of altered protein expression ranked upon metric score (right). **(b)** Corresponding heatmap of the glutathione metabolic process pathway (n=3). **(c)** An unsupervised hierarchical clustering of the proteins of the GO term "Glutathione Metabolic Process".

Figure S2

A

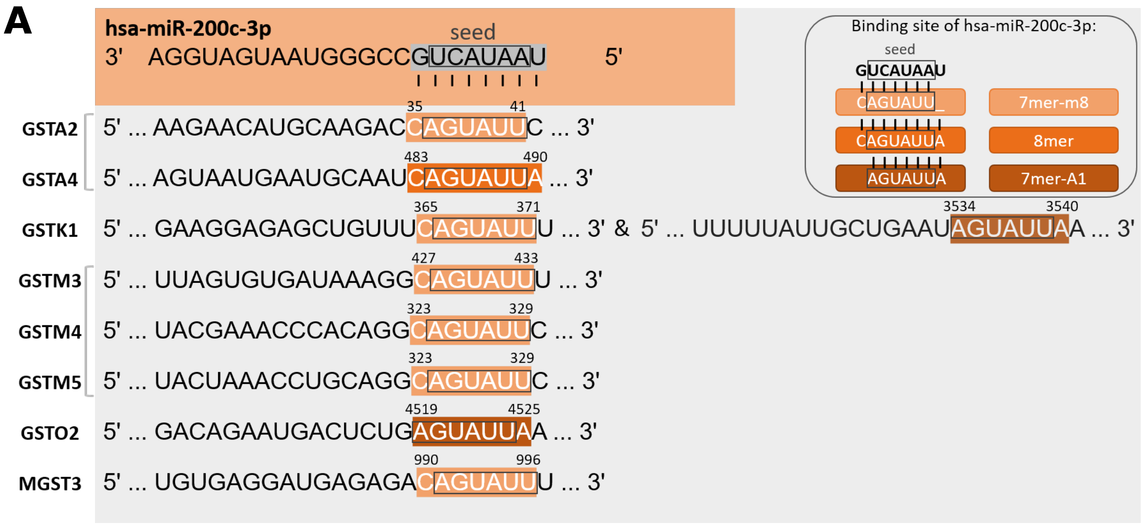

B

Gene Name	MCF7 KO 200c	MCF7 Tripz 200c sponge	MDA-MB 231 Tripz 200c	Binding Site	Results
GSTM1	n.d.	n.d.	↓	-	∞ (regulated even if no predicted binding site)
GSTM2	n.d.	n.d.	ns	-	✓
GSTM3	↑	↑	↓	+	✓
GSTM4	↑	↑	ns	+	∞ (not significant in MDA-MB 231 Tripz 200c)
GSTM5	n.d.	n.d.	n.d.	+	✓
GSTA1	n.d.	n.d.	n.d.	-	✓
GSTA2	n.d.	n.d.	n.d.	+	✓
GSTA3	n.d.	n.d.	n.d.	-	✓
GSTA4	n.d.	n.d.	n.d.	+	✓
GSTA5	n.d.	n.d.	n.d.	-	✓
GSTK1	↑	↑	↑	+	∞ (differently regulated in MDA-MB 231 Tripz 200c)
MGST1	↑	↑	n.d.	-	∞ (regulated even if no predicted binding site)
MGST2	ns	n.d.	n.d.	-	✓
MGST3	↑	↑	n.d.	+	✓
LTC4S	n.d.	n.d.	n.d.	-	✓
FLAP	n.d.	n.d.	ns	-	✓
GSTO1	↑	↓	↓	-	∞ (differently regulated in MCF7 Tripz 200c sponge and regulated even if no predicted binding site)
GSTO2	↑	ns	↑	+	∞ (differently regulated in MDA-MB 231 Tripz 200c and not significant in MCF7 Tripz 200c sponge)
GSTP1	n.d.	n.d.	↓	-	∞ (regulated even if no predicted binding site)
GSTT1	ns	ns	n.d.	-	✓
GSTT2	↑	↑	n.d.	-	∞ (regulated even if no predicted binding site)
GSTZ1	ns	ns	↓	-	∞ (regulated even if no predicted binding site)

C

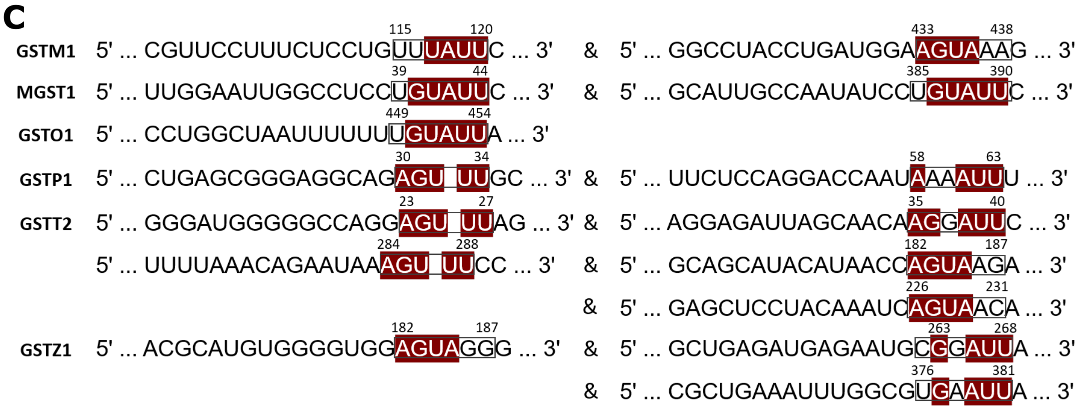

D

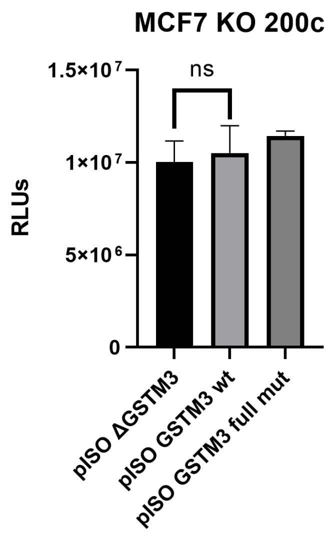

Figure S2: *In silico* miRNA-mRNA seed-site interaction. (a) 3`UTR sequences of GSTs with a predicted target site for hsa-miR-200c-3p. The seed region and complementary region are framed. Numbers indicate position within the 3`UTR sequence. Illustration of the 7mer-m8, 8mer and 7mer-A1 is shown on the right. Adapted from TargetScan. **(b)** Comparison of the regulation of all GSTs in the three hsa-miR-200c expression systems. Results, indicated with a check mark, verify the predicted miRNA-mRNA seed-site interaction. Further evaluation of the interaction is needed if the infinity symbol is displayed. n.d. = not detected, ns = not significant **(c)** Possible target sites for hsa-miR-200c-3p seed region. Framed sequences display possible complementary regions. Dark red highlighted nucleotides show a complementary match with the hsa-miR-200c-3p seed region. Sequences are adapted from TargetScan. **(d)** Luciferase assay of different GSTM3 3`UTR reporter plasmids co-transfected with a scrambled siRNA in MCF7 KO 200c cells. One representative diagram is shown. A two-tailed Student's t-test for pISO ΔGSTM3 and pISO GSTM3 wt was performed. Values are displayed as mean with SD.

Figure S3

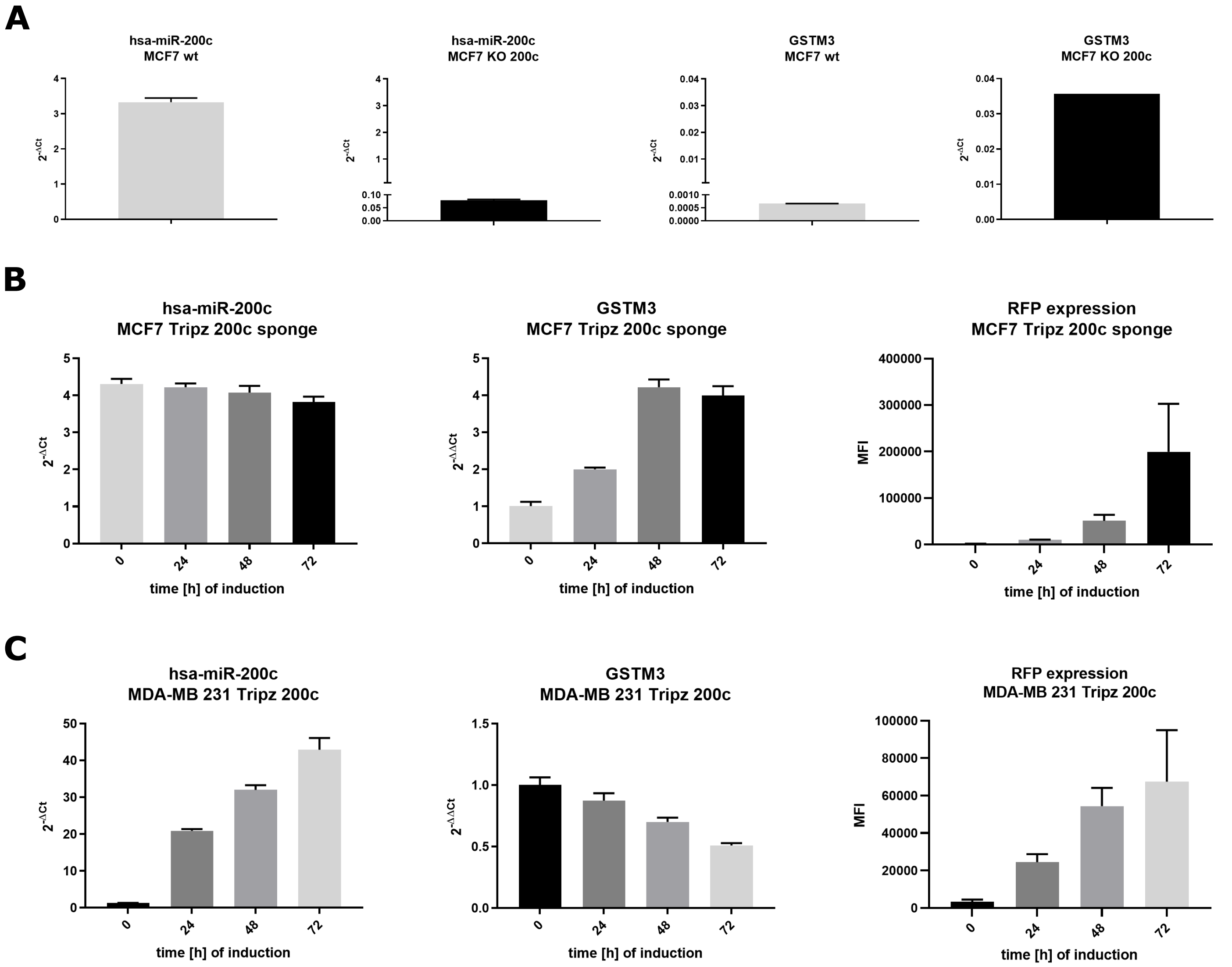

Figure S3. Characterization of hsa-miR-200c expression systems. (a) qRT-PCR analysis of hsa-miR-200c and GSTM3 expression in MCF7 wt and KO 200c cells. Quantitative RT-PCR analysis of hsa-miR-200c and GSTM3 expression in **(b)** MCF7 Tripz 200c sponge cells and **(c)** in MDA-MB 231 Tripz 200c cells, each induced with 5 µg/ml doxycycline and FACS analysis of RFP expression of the same cells displayed as MFI. One representative replicate out of three is displayed. Values are displayed as mean with SD.

Figure S4

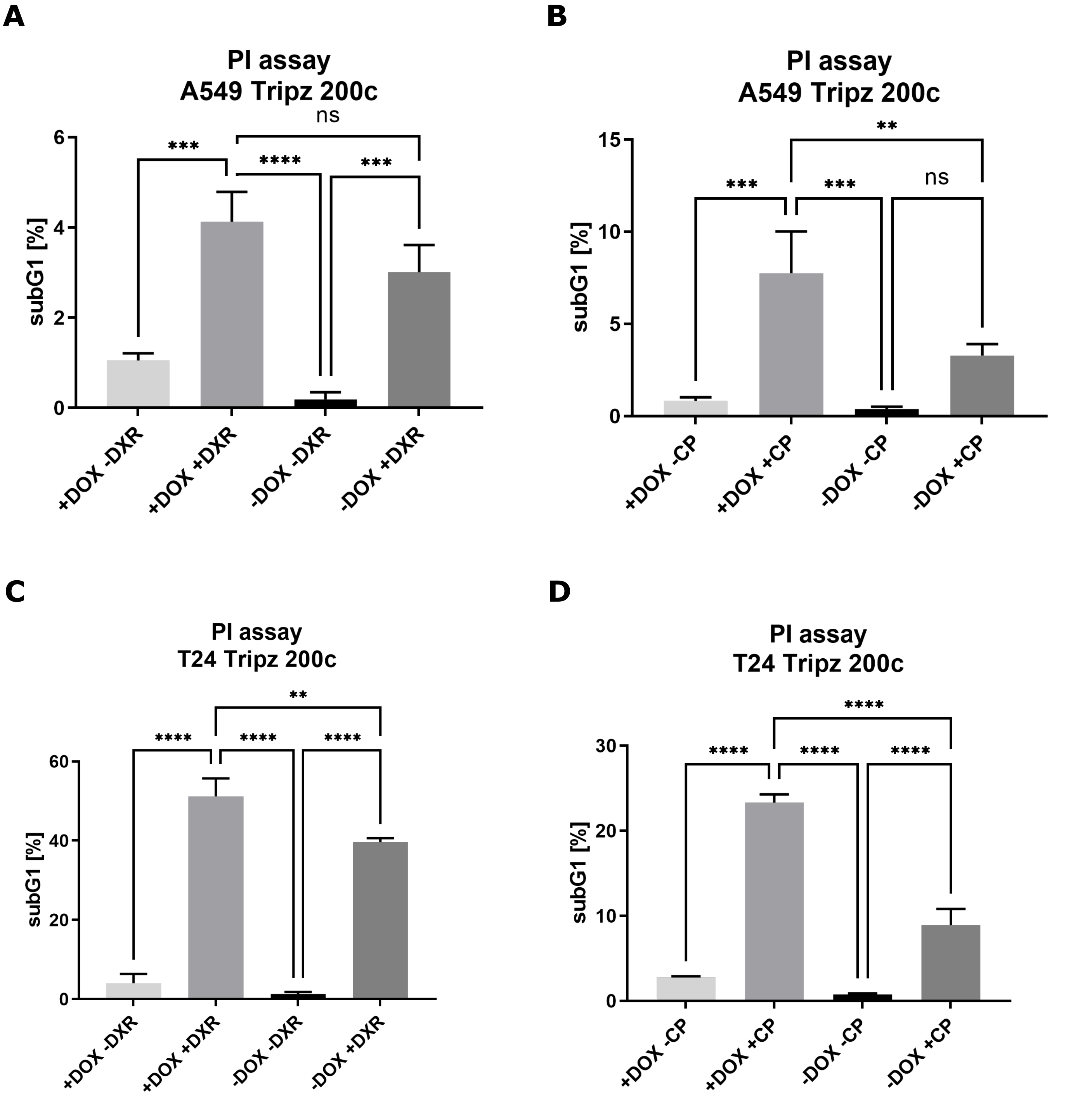

Figure S4. Effect of hsa-miR-200c on cell death in two additional cancer types in combination with chemotherapeutic treatment. Analysis of subG1 population in **(a, b)** A549 lung cancer cells in combination with doxorubicin (DXR) or cisplatin (CP) treatment and **(c, d)** T24 bladder cancer cells in combination with DXR or CP treatment using propidium iodide assay. Both cell lines were 72 hours pre-induced with DOX or not (-DOX). An ordinary one-way ANOVA with Tukey's multiple comparison test was performed for statistics. ** $p < 0.01$, *** $p < 0.001$, ****$p < 0.0001$. One representative diagram out of three is displayed. Values are displayed as mean with SD.

Figure S5

A

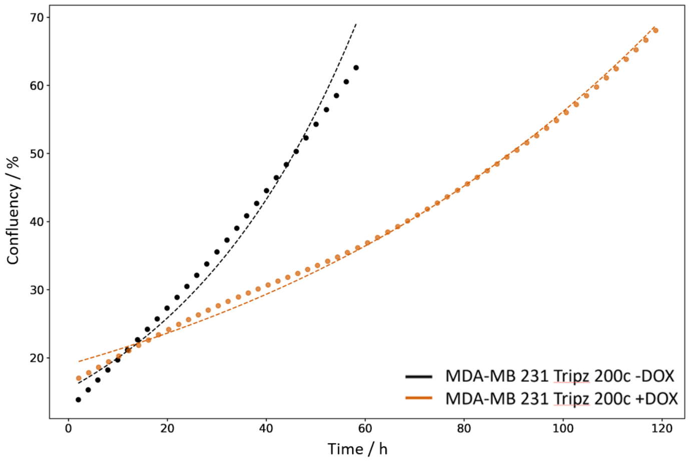

Period of time for confluency change from 30% to 60%	
MDA-MB 231 Tripz 200c -DOX	32.2 h
MDA-MB 231 Tripz 200c +DOX	68.38 h

Figure S5. Proliferation curves of hsa-miR-200c positive or negative MDA-MB 231 cells. Proliferation of **(a)** MDA-MB 231 Tripz 200c with doxycycline induction every 48 to 72 hours. Comparison of proliferation slopes and doubling times in hsa-miR-200c expressing (+DOX, orange curve) and hsa-miR-200c depleted (-DOX, black curve) cells. One representative diagram out of three is presented.

Figure S6

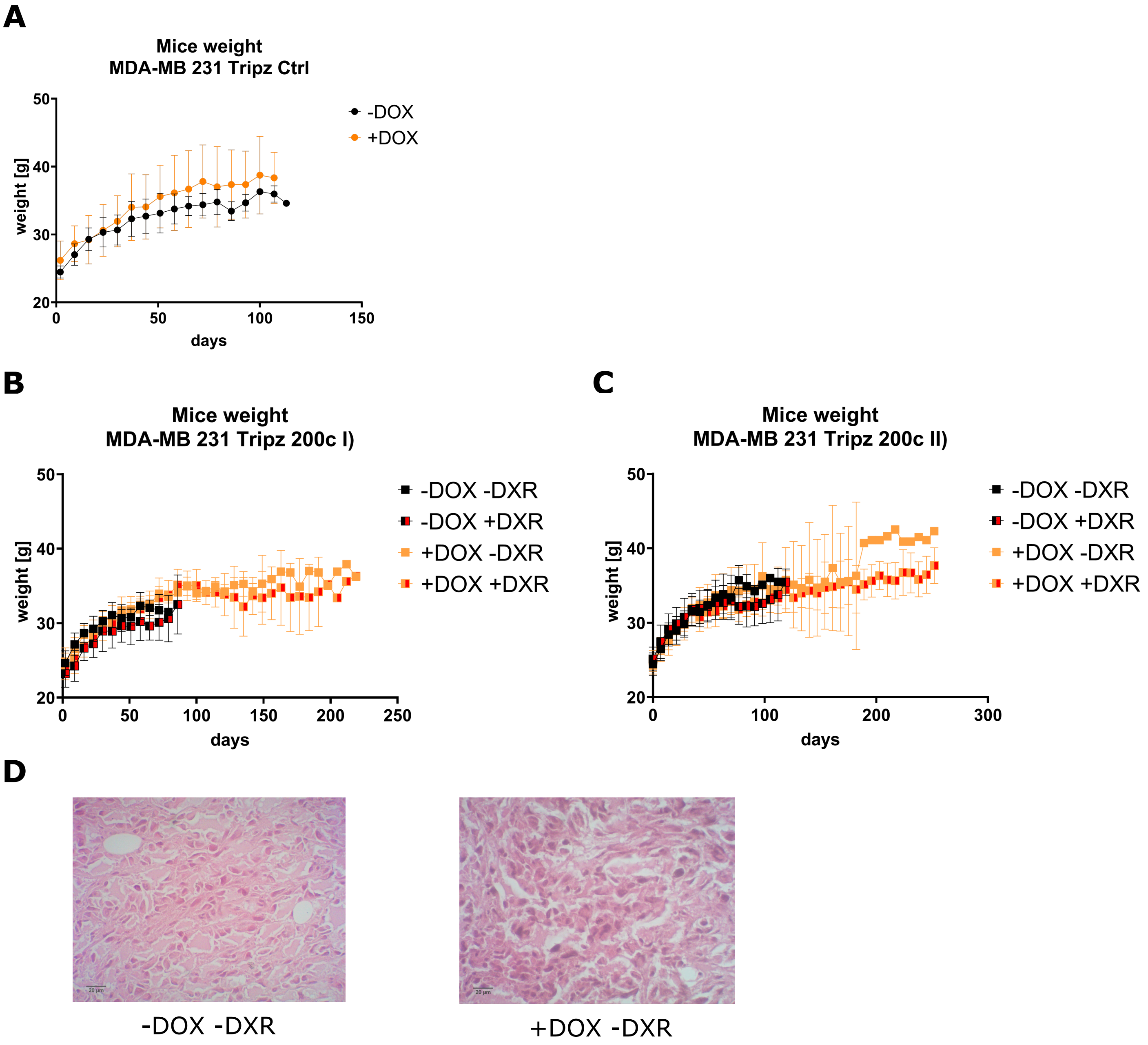

Figure S6. Animal welfare monitoring of the different xenograft mouse models and histological analysis of hsa-miR-200c positive and negative tumors. (a) Weekly body weight mean of mice bearing control tumors (MDA-MB 231 Tripz Ctrl) and fed with regular (black curves) or doxycycline (DOX, orange curves) diet (n=5). **(b)** Body weight mean displayed once a week of mice with hsa-miR-200c positive (MDA-MB 231 Tripz 200 +DOX, orange curves) or negative (-DOX, black curves) tumors and additional chemotherapeutic treatment (n=5, red filled squares) related to animal experiment I) Treatment of hsa-miR-200c positive and negative tumors. **(c)** Mean body weight (displayed once a week) of mice with initial hsa-miR-200c negative tumors and their subsequent treatment with either doxorubicin (DXR, black curve and red filled squares) or miRNA-200c (orange curve) or their combination (orange curve and red filled squares) related to animal experiment II) Single or double treatment of hsa-miR-200c negative tumors, (n=10 per group). Body weight graphs do not terminate when mice were euthanized. **(d)** H&E staining of hsa-miR-200c negative (-DOX) or positive (+DOX) tumors without DXR treatment. Values are displayed as mean with SD.

Figure S7

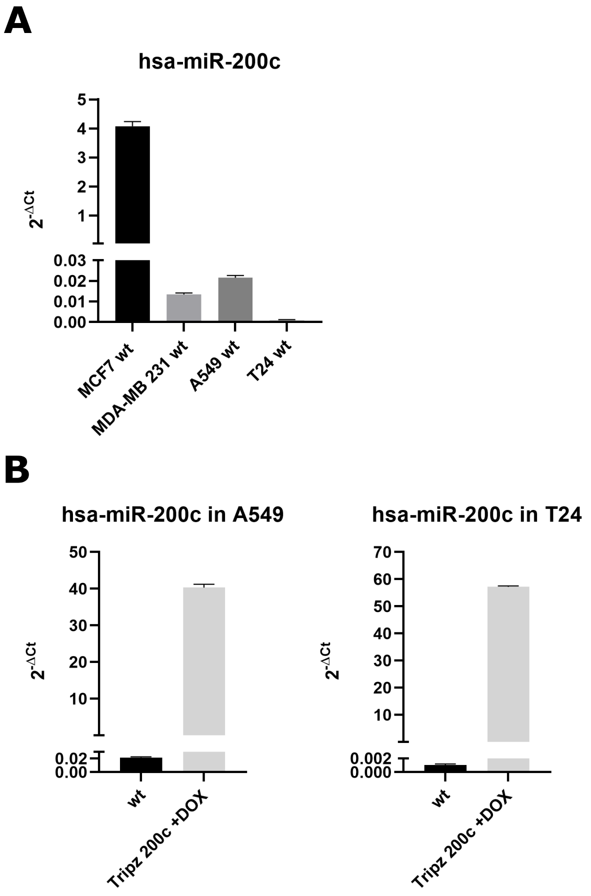

Figure S7. hsa-miR-200c expression profile. (a) qRT-PCR analysis of hsa-miR-200c expression in the wt cell lines. **(b)** Induction of hsa-miR-200c in A549 Tripz 200c (left) and T24 Tripz 200c (right) upon doxycycline application (5 µg/ml) compared to the corresponding wt cell line. Values are displayed as mean with SD.

Figure S8

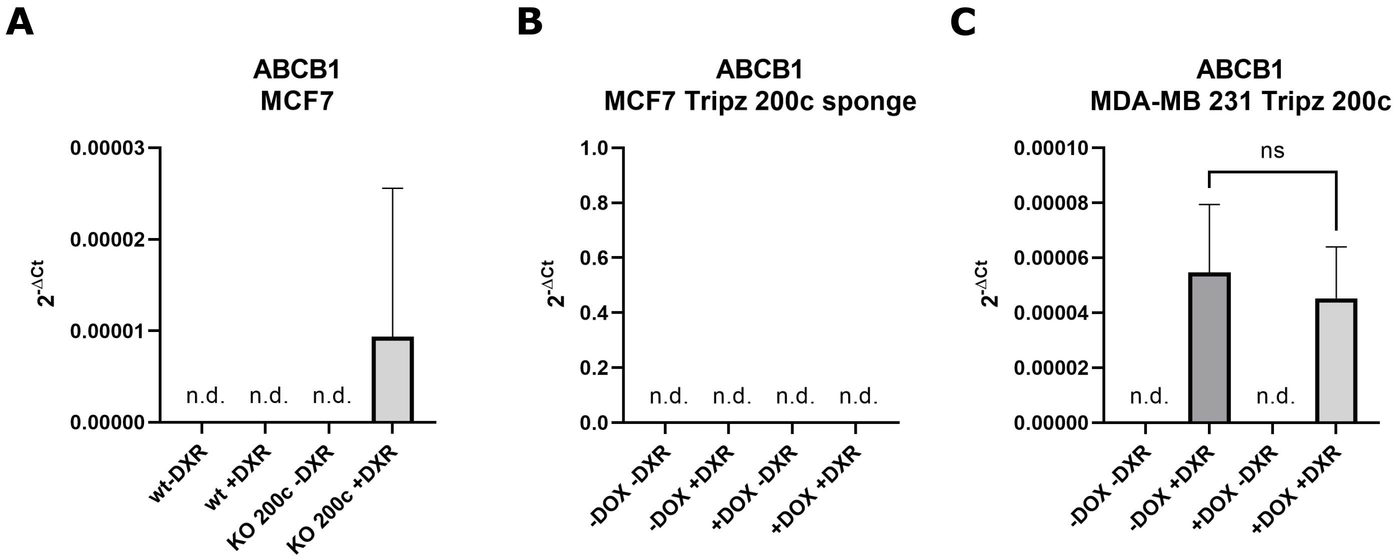

Figure S8. hsa-miR-200c expression and the abundance of ABCB1. Expression of ABCB1 analyzed with qRT-PCR in the **(a)** MCF7 wt and KO 200c (treatment with 5 µM of DXR for 6 hours), **(b)** the MCF7 Tripz 200c sponge (72 hours DOX pre-induced and treated with 0.1 µM DXR for 24 hours) and **(c)** the MDA-MB 231 Tripz 200c cell system (72 hours DOX pre-induced and subsequently treated with 0.6 µM DXR for 24 hours). Ct values of 35 and more are uncertain values and therefore were excluded from the analysis and termed as not detected (n.d.). ns = not significant. Values are displayed as mean with SD.

Figure S9

**Kaplan-Meier plot for hsa-miR-200c**	
miRNA: miRpower for breast cancer	
miRNA ID	hsa-miR-200c
Survival	Overall survival
Split patients by	auto select best cutoff
Follow up threshold	60 months, censore at threshold
Dataset	TCGA
ER status	IHC and gene chip: all
PGR status	IHC: all
HER2 status	IHC and gene chip: all
Molecular subtype	TNBC
Grade	all
Lymph node status	all
Restrict analysis to selected cohorts	do not use cohorts
**Kaplan-Meier plot for GSTM3**	
mRNA gene chip for breast cancer	
Affy ID	202554_s_at
Survival	Relapse free survival (RFS)
Split patients by	median
Follow up threshold	60 months, censore at threshold
Probe set option	only JetSet best probe set
ER status	IHC and array: all
PR status	IHC: all
HER2 status	array: all
Subtype - StGallen	basal
Subtype - PAM50	all
Lymph node status	all
Grade	all
TP53 status	all
Pietenpol subtype	all
Use earlier release of the database	all
Use following dataset for the analysis	all
Quality control - remove redundant samples	checked
Quality control - array quality control	exclude biased arrays
Quality control - proportional hazards assumption	checked
Cohorts	patients with following systemic treatment
Endocrine therapy	any
Chemotherapy	neoadjuvant only

Figure S9: Overview on the parameters entered into the Kaplan-Meier Plotter for the analysis of hsa-miR-200c and GSTM3 in breast cancer patients.

Figure S10

For Figure 4B:

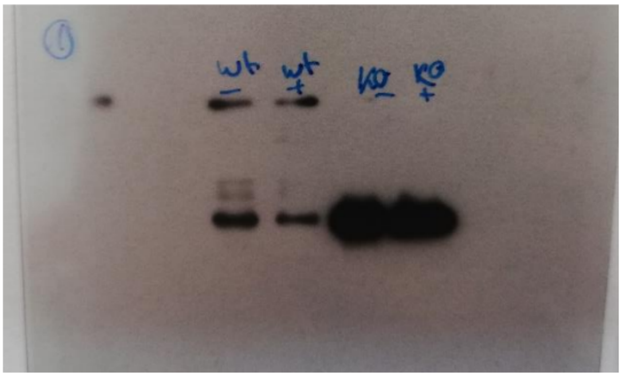

GSTM3

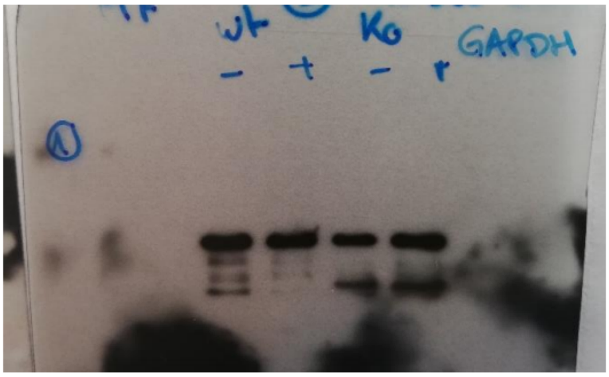

GAPDH

For Figure 4D:

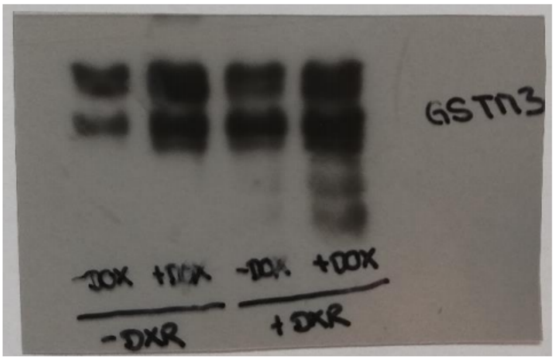

GSTM3

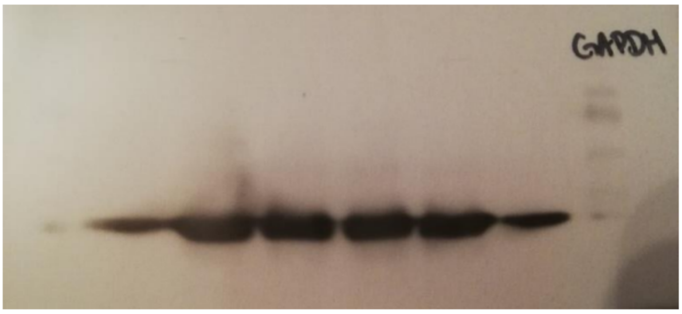

GAPDH

For Figure 4F:

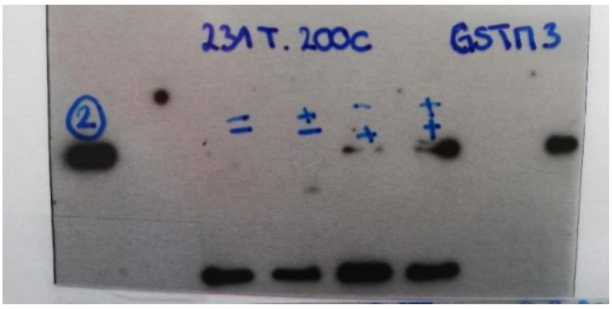

GSTM3

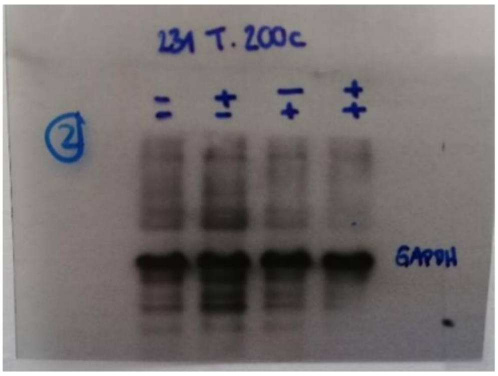

GAPDH

Figure S10: Uncropped blots of western blots presented in Figure 4B,D,F

Table S1

	MCF7 wt				MCF7 KO		
gene	Ct value 1	Ct value 2	Ct value 3	gene	Ct value 1	Ct value 2	Ct value 3
mu family				mu family			
GSTM1	38.19	37.92		GSTM1			
GSTM2	36.84	36.64	37.84	GSTM2	35.08	35.05	35.72
GSTM3	28.2	28.18	28.18	GSTM3	22.68	22.68	22.68
GSTM4	26.85	26.88	26.91	GSTM4	24.46	24.44	24.43
GSTM5	36.83	35.62	36.15	GSTM5	35.9	35.1	35.25
GAPDH	17.63	17.62	17.6	GAPDH	17.91	17.86	17.84
alpha and kappa family				alpha and kappa family			
GSTA1	36.84		38.74	GSTA1	35.08	36.44	34.81
GSTA2	37.27	36.94	35.99	GSTA2	34.45	34.43	34.92
GSTA3	40	36	36.37	GSTA3	32.77	32.54	32.8
GSTA4	39.64	39.55	39.36	GSTA4	38.31	39.04	39.28
GSTA5	37.97	38.19	36.64	GSTA5	33.24	33.44	33.49
GSTK1	24.83	24.94	24.87	GSTK1	24.27	24.27	24.26
GAPDH	17.72	17.54	17.54	GAPDH	17.89	17.88	17.85
MAPEG family part 1				MAPEG family part 1			
MGST1	25.17	24.96	25.04	MGST1	23.26	23.42	23.41
MGST3	25.18	25.55	25.21	MGST3	23.79	23.65	23.74
GAPDH	17.72	17.54	17.54	GAPDH	17.89	17.88	17.85
MAPEG family part 2				MAPEG family part 2			
LTC4S	37.73	39.12	40	LTC4S	38.46		
FLAP	40			FLAP	38.35	40	
GAPDH	18.03	18.05	18.14	GAPDH	17.97	18.04	18.1
MAPEG family part 3				MAPEG family part 3			
MGST2	33.16	32.93	31.93	MGST2	32.92	34.35	34.08
GAPDH	17.63	17.62	17.6	GAPDH	17.91	17.86	17.84
omega, pi, theta and zeta family				omega, pi, theta and zeta family			
GSTO1	32.11	32.36	32.16	GSTO1	32.12	31.99	32.18
GSTO2	25.17	25.26	25.24	GSTO2	25.32	25.35	25.42
GSTP1	36.15	36.56	35.53	GSTP1	37.86	40	40
GSTT1	26.52	26.52	26.37	GSTT1	26.65	26.66	26.8
GSTT2	32.6	32.87	32.2	GSTT2	31.88	31.59	31.26
GSTZ1	23.71	23.81	23.58	GSTZ1	24.14	24.16	24.13
GAPDH	17.72	17.54	17.54	GAPDH	17.89	17.88	17.85

	MCF7 Tripz 200c sponge -DOX				MCF7 Tripz 200c sponge +DOX		
gene	Ct value 1	Ct value 2	Ct value 3	gene	Ct value 1	Ct value 2	Ct value 3
mu family				mu family			
GSTM1				GSTM1			
GSTM2	36.16	36.62	36.16	GSTM2	33.96	34	33.73
GSTM3	29.36	29.33	29.34	GSTM3	28.03	28	28
GSTM4	27.21	27.17	27.25	GSTM4	26.76	26.76	26.66
GSTM5				GSTM5			
GAPDH	19.13	19.25	19.24	GAPDH	19.27	19.22	19.11
alpha and kappa family				alpha and kappa family			
GSTA1	40	40	40	GSTA1	40	40	40
GSTA2	35.8	36.58	36.84	GSTA2	35.2	36.19	35.49
GSTA3				GSTA3		40	
GSTA4			40	GSTA4			
GSTA5	36.36	37	37.62	GSTA5	36.84	35.96	36.09
GSTK1	24.9	24.94	25	GSTK1	24.26	24.25	24.3
GAPDH	19.13	19.25	19.24	GAPDH	19.27	19.22	19.11
MAPEG family part 1				MAPEG family part 1			
MGST1	27.05	26.87	27.03	MGST1	26.54	26.67	26.35
MGST2	37.27	37.09	37.17	MGST2	36.79	37.04	36.72
MGST3	25.32	25.4	25.56	MGST3	24.95	24.87	24.79
LTC4S		39.11	38.43	LTC4S	40	38.03	40
GAPDH	19.13	19.25	19.24	GAPDH	19.27	19.22	19.11
MAPEG family part 2				MAPEG family part 2			
FLAP		40		FLAP			
GAPDH	18.99	19.11	19.13	GAPDH	19.15	19.14	19.13
omega, pi, theta and zeta family				omega, pi, theta and zeta family			
GSTO1	33.67	33.52	33.44	GSTO1	33.94	34.05	34.37
GSTO2	25.75	25.8	25.9	GSTO2	25.9	25.84	25.87
GSTP1	35.46	35.98	37.31	GSTP1	36.74	36.3	35.94
GSTT1	25.6	25.79	25.73	GSTT1	25.13	25.9	25.49
GSTT2	35.43	35.51	35.66	GSTT2	34.95	34.66	34.93
GSTZ1	23.78	23.53	23.67	GSTZ1	23.64	23.68	23.73
GAPDH	18.99	19.11	19.13	GAPDH	19.15	19.14	19.13

	MDA-MB 231 Tripz 200c -DOX				MDA-MB 231 Tripz 200c +DOX		
gene	Ct value 1	Ct value 2	Ct value 3	gene	Ct value 1	Ct value 2	Ct value 3
mu family				mu family			
GSTM1	26.85	26.87	26.92	GSTM1	27.51	27.53	27.62
GSTM2	28.25	28.29	28.91	GSTM2	29.05	28.86	29.12
GSTM3	27.84	27.78	27.82	GSTM3	28.37	28.28	28.25
GSTM4	25.86	26	26.03	GSTM4	26.23	26.16	26.23
GSTM5	37.65	37.11	40	GSTM5	37.61	38.18	
GAPDH	19.6	19.47	19.46	GAPDH	19.74	19.68	19.66
alpha and kappa family				alpha and kappa family			
GSTA1				GSTA1			
GSTA2				GSTA2			
GSTA3	40	40	40	GSTA3	38.53	40	37.25
GSTA4	37.42	36.76	37.28	GSTA4	40	40	37.63
GSTA5	38.38	40		GSTA5	40	40	
GSTK1	24.88	24.87	24.86	GSTK1	24.67	24.66	24.67
GAPDH	19.42	19.24	19.01	GAPDH	19.26	19.34	19.16
MAPEG family part 1				MAPEG family part 1			
MGST1				MGST1			
MGST2				MGST2			
MGST3				MGST3			
GAPDH	19.42	19.24	19.01	GAPDH	19.26	19.34	19.16
MAPEG family part 2				MAPEG family part 2			
LTC4S		40	40	LTC4S			
FLAP	31.89	31.98	31.9	FLAP	32.21	32.43	32.34
GAPDH	19.34	19.46	19.46	GAPDH	19.63	19.66	19.68
omega, pi, theta and zeta family				omega, pi, theta and zeta family			
GSTO1	29.73	29.67	29.54	GSTO1	30	30.09	30.06
GSTO2	30.8	30.79	30.76	GSTO2	29.98	30.11	30.08
GSTP1	23.23	23.2	23.25	GSTP1	23.62	23.62	23.82
GSTT1				GSTT1			
GSTT2				GSTT2			
GSTZ1	24.13	24.06	23.87	GSTZ1	24.67	24.6	24.65
GAPDH	19.42	19.24	19.01	GAPDH	19.26	19.34	19.16

Table S1: Raw Ct values of qRT-PCR analysis of all glutathione S-transferases in the different hsa-miR-200c expression systems. Table shows raw Ct values in triplicates for each sample gene. Every GST family is displayed with its corresponding housekeeper (GAPDH) values. Undetectable Ct values (Ct> 40) are not displayed. For the analysis the $2^{-\Delta Ct}$ method was used. Ct values between 35 and 40 are uncertain values and therefore termed as not detected (n.d.) in Figure 3B-D.
